# Direct Air Capture and Integrated Conversion of Carbon Dioxide into Cyclic Carbonates with Basic Organic Salts

**DOI:** 10.1021/acssuschemeng.3c00890

**Published:** 2023-06-23

**Authors:** Marcileia Zanatta, Eduardo García-Verdugo, Victor Sans

**Affiliations:** † Institute of Advanced Materials (INAM), Univesitat Jaume I, Avda Sos Baynat s/n, Castellón 12071, Spain; ‡ Departamento de Química Inorgánica y Orgánica, Grupo de Química Sostenible y Supramolecular Universidad Jaume I, E-12071 Castellón, Spain

**Keywords:** ionic liquids, atmospheric air, direct air capture and conversion, halohydrin, biomass, carbon dioxide

## Abstract

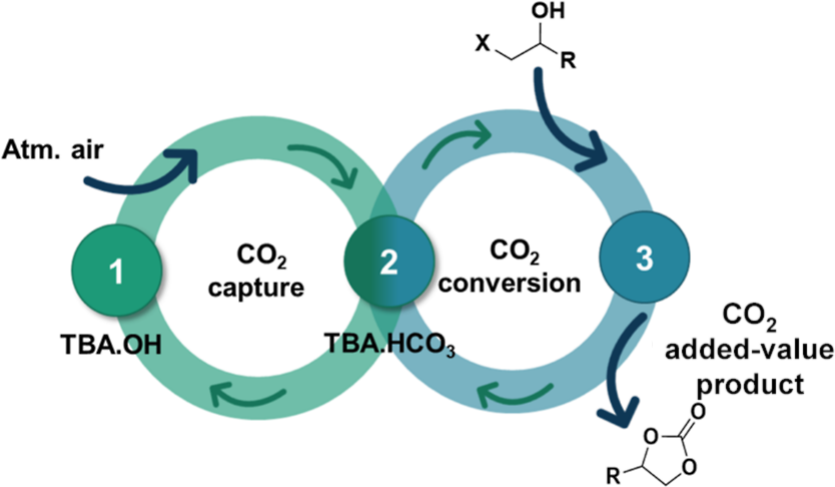

Direct air capture and integrated conversion is a very attractive strategy to reduce CO_2_ concentration in the atmosphere. However, the existing capturing processes are technologically challenging due to the costs of the processes and the low concentration of CO_2_. The efficient valorization of the CO_2_ captured could help overcome many techno-economic limitations. Here, we present a novel economical methodology for direct air capture and conversion that is able to efficiently convert CO_2_ from the air into cyclic carbonates. The new approach employs commercially available basic ionic liquids, works without the need for sophisticated and expensive co-catalysts or sorbents and under mild reaction conditions. The CO_2_ from atmospheric air was efficiently captured by IL solution (0.98 molCO_2_/mol_IL_) and, subsequently, completely converted into cyclic carbonates using epoxides or halohydrins potentially derived from biomass as substrates. A mechanism of conversion was evaluated, which helped to identify relevant reaction intermediates based on halohydrins, and consequently, a 100% selectivity was obtained using the new methodology.

## Abstract 2

Capture of CO_2_ direct from the atmospheric air and transformation into added-value products under mild conditions.

## Introduction

Carbon dioxide is the largest contributor to green house gas (GHG) emissions, responsible for the global warming and the climate change.^1^ Direct air capture of CO_2_ (DAC) has attracted attention among various proposed solutions to reduce the concentration of CO_2_ in the atmosphere. This is a very challenging process due to the relatively low concentration of CO_2_ in the atmosphere. Thus, chemisorbent materials (e.g., amines or hydroxides) have proven to be much more effective for the DAC processes.^2^ This renders thermodynamically stable adducts (e.g., carbamates and carbonates), which require large amounts of energy to recover the CO_2_ or low added-value products (e.g., CaCO_3_).^2−4^ A finely balanced interaction between the CO_2_ and the sorbents is required to enable its capture and, subsequently, transformation into added-value products.^2,5,6^ Generally, direct air capture and conversion (DACC) processes are limited to thermodynamic stability of CO_2_, which puts up a major synthetic challenge for its conversion under mild conditions.^7,8^

**Figure 1:**
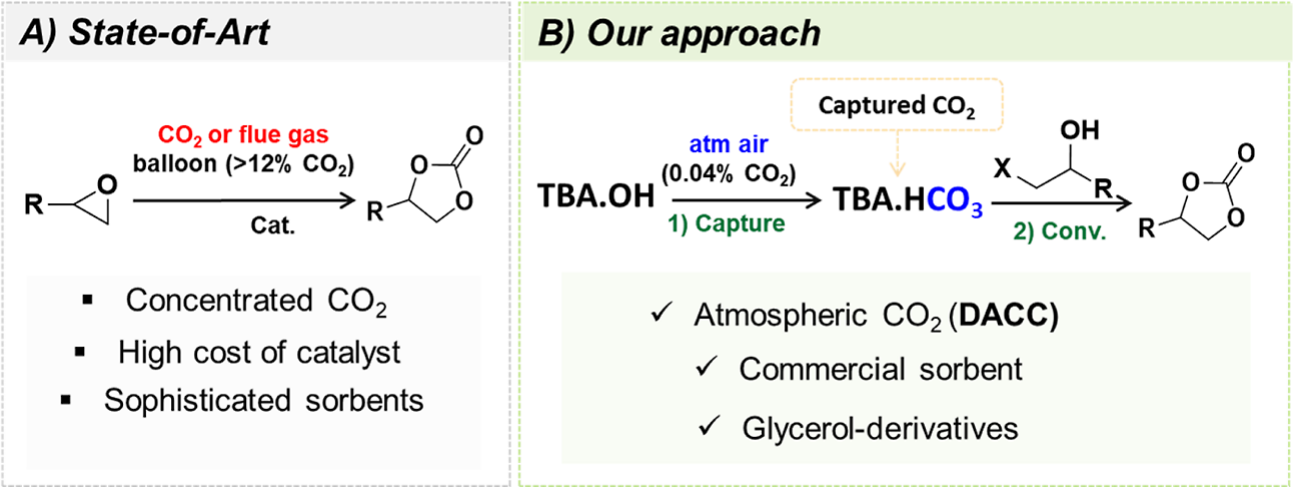
General low-pressure CCU methodologies comparing the state of the art (a) and this work (b).

The production of cyclic carbonates from CO_2_ is very interesting from a sustainability point of view due to the high atom economy of the reaction, since it presents complete atomic efficiency. Cyclic carbonates are important industrial products that have been widely applied as polar aprotic solvents, organic synthesis intermediates, electrolytes in lithium-ion batteries, cosmetic formulations, and monomers, contributing in over 90 billion to the EU economy within these sectors.^9,10^ Great efforts have been focused on the development of efficient and sustainable catalytic systems that work under relatively mild conditions. Successful examples using different catalytic systems under 1 atm of concentrated CO_2_ have been already reported for the CO_2_ cycloaddition to epoxides^9,11,12^ and/or to halohydrins.^13,14^ However, only few examples of CO_2_ transformation using diluted sources of CO_2_, such as atmospheric air (0.04% of CO_2_) or flue gases (12–15% of CO_2_) have been reported.^15−18^ For example, indium tribromide (InBr_3_) combined with tetrabutylammonium bromide (TBAB) as co-catalyst showed moderate conversions (>80%) into cyclic carbonates under sub-atmospheric pressures of CO_2_ (flue gas balloon).^17^ Following a similar methodology (Figure 1A), protic ionic liquids (ILs) have been used to produce cyclic carbonates from flue gas with good catalytic activity (78–99%).^15^ Comparing the reaction using concentrated CO_2_ with the reaction using flue gas, longer reaction times were required to obtain similar conversion and the reaction time increased from 6 to 8 h to 32–72 h in flue gas.^15^ The concentration of CO_2_ in the flue gas compared to air is 300–400 times, thus indicating the magnitude of the challenge for the DACC process.

The development of a single material that combines sorption and catalytic abilities to work simultaneously or sequentially for both the capture and conversion of CO_2_ is very attractive but challenging. Drawing CO_2_ directly from the atmosphere could have clear climate benefits, but capturing the gas from the air is extremely expensive, as is the sequential conversion.^19^ Until now, it has been required to use sophisticated and, thus, expensive sorbents and catalysts. For instance, in a recent pioneering work, Mg(II)-based MOFs demonstrated an efficient catalyst for directly converting CO_2_ from the atmospheric air into cyclic carbonates under mild conditions (60 °C, 48 h, balloon loaded with air), resulting in 92% of conversion for epichlorohydrin (ECH).^16^ However, the use of epoxide substrates can be considered dangerous from the industrial point of view due to its flammability.^20^ In addition, the effect on the CO_2_ concentration using diluted sources in the mechanism of reaction is not well established in the literature.^15^

Herein, we report an economical methodology to integrate the direct capture of CO_2_ with air and their efficient and selective conversion into cyclic carbonates without using epoxides as substrates (Figure 1B). The method employed uses metal-free, low-cost commercially available organic salts under mild reaction conditions (atm. air, 40 °C), a considerably short reaction time (16 h) and works with a variety of solvents and substrates. In a first attempt, ECH was used to evaluate the conditions for the transformation of the captured CO_2_ and a reaction mechanism was proposed, with the formation of halohydrins as intermediate species. From them, glycerol-derivatives and other halohydrin were used to selectively convert atmospheric CO_2_ into cyclic carbonate, avoiding the presence of epoxide.

## Results and Discussion

Capturing CO_2_ is a highly challenging process that typically requires high concentration of CO_2_ to generate steep concentration gradients and adequate sorbents like ILs with basic and hygroscopic anions (e.g., acetate, imidazolate, and hydroxide), which form HCO_3_^–^.^21,22^ Meanwhile other studies have demonstrated the potential of hydroxy-based solutions (alkali or tertiary amines) to capture CO_2_ directly from the air to form RHCO_3_, even though large energy penalties were required to reverse the sorption process due to the stability of the adducts formed.^3,8^ Here, two hypotheses are considered to design the work: (i) a solution of organic hydroxide salts will be able to capture CO_2_ directly from the air forming concentrated solutions of HCO_3_^–^; and (ii) the captured CO_2_ (HCO_3_^–^) sequentially transformed under mild conditions, thus reducing the energy costs compared to the use of a carbamate as a substrate.^4^ This effective decoupling of the capture and activation could lead to increased efficiency of the capture process by generating highly concentrated HCO_3_^–^ solutions in the first place that, subsequently, increase conversion kinetics. The new approach can be observed in Figure 1, where the CO_2_ is previously bubbled into the IL solution (step 1: CO_2_ capture), and the substrate is added in the second stage (step 2: CO_2_ conversion). By employing this strategy, the CO_2_ concentration is locally enhanced at the reaction site, which helps to overcome the kinetic and thermodynamic barriers to its transformation.

**Figure 2:**
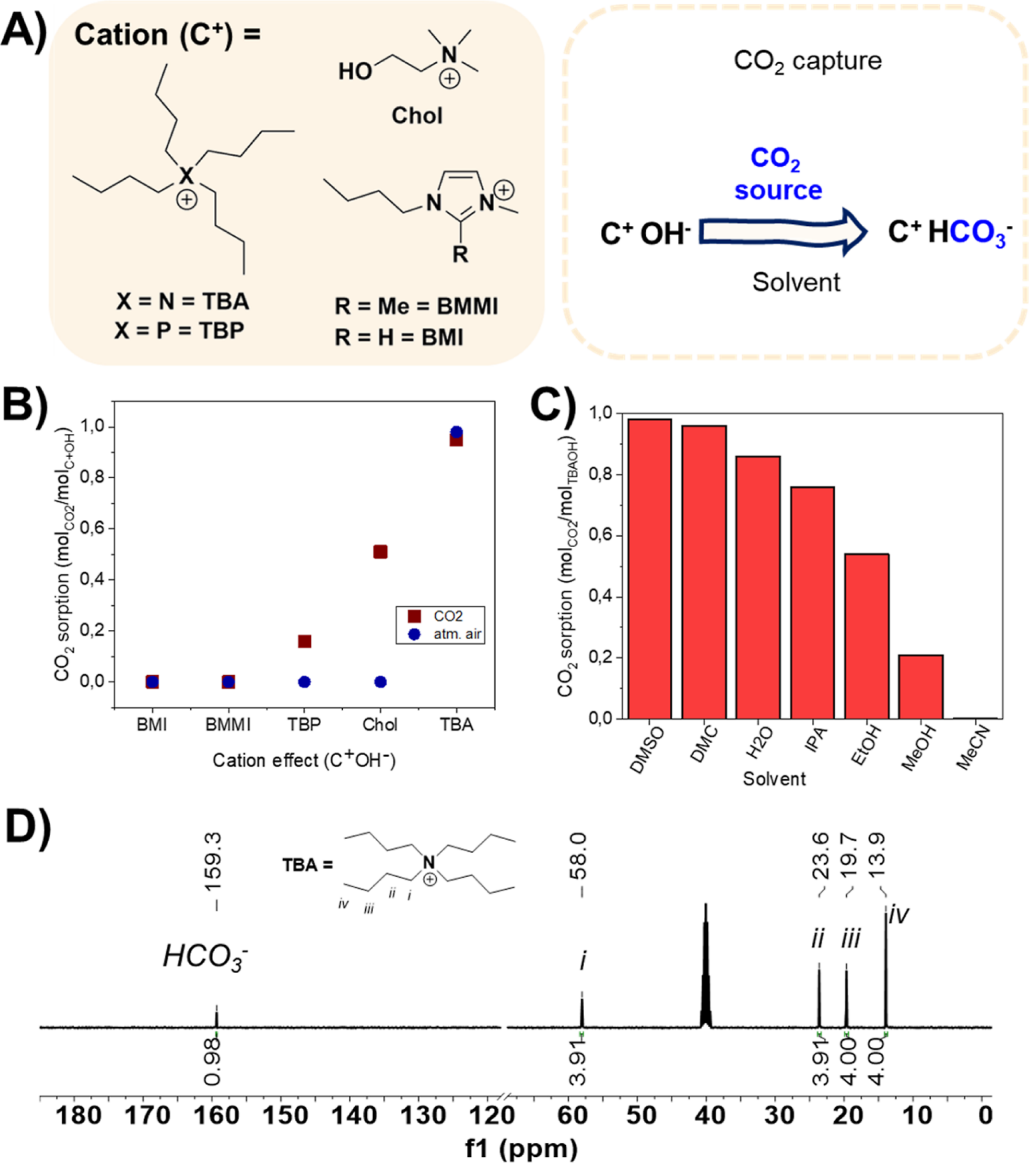
CO_2_ capture evaluation. (A) Chemical structure of cations used in this work. General sorption conditions: Bubbling CO_2_ into 1 M solution, 25 °C for 15 min (CO_2_), or 16 h (atmospheric air). (B) Using different CO_2_ sources and hydroxide-based salts (C^+^OH^–^). (C) Quantitative ^13^C NMR spectra (100 MHz) of TBA.OH (1 M in DMSO-*d*_6_) after 16 h of bubbling atmospheric air.

To check the hypothesis, the capture process using concentrated CO_2_ and atmospheric air was evaluated (Figure 1B), step (1), using different hydroxide organic salts (Figure 2A,B) and different solvents (Figure 2C). The sorption capacity using concentrated CO_2_ presented the following order: tetrabutylphosphonium hydroxide (TBP.OH) (0.16 mol_CO2_/mol_sorb_) < cholinium hydroxide (Chol.OH) (0.51 mol_CO2_/mol_sorb_) < tetrabutylammonium hydroxide (TBA.OH) (0.95 mol_CO2_/mol_sorb_) (see Supporting Information, Table S1, Figures S8–S9). It is important to punctuate that the imidazolium-based ILs used here, 1-^*n*^butyl-3-methylimidazolium hydroxide (BMI.OH) and 1-^*n*^butyl-2,3-dimethyl-imidazolium hydroxide (BMMI.OH), both degraded in DMSO and, therefore, do not present any sorption capacity. When atmospheric air was employed, a remarkable sorption capacity of 0.98 mol_CO2_/mol_sorb_ was obtained using TBA.OH (Figure 2D). The other salts do not present any capacity to capture CO_2_ directly from the air.

Further, the sorption capacity of TBA.OH using other solvents was evaluated. The solvent employed demonstrated to have a profound influence on the capture and conversion ability. The best results for capture and conversion were obtained with dimethylsulfoxide (DMSO), showing 0.98 mol_CO2_/mol_TBAOH_, and in dimethyl carbonate (DMC) with 0.95 mol_CO2_/mol_TBAOH_ (Figure 2C, Table S2 and Figures S10–S11), which is considered a green solvent.^23^ Alcohols showed moderate to good sorption capacity yielding different RCO_3_^–^ species (0.21 mol_CO2_/mol_TBAOH_ in MeOH, 0.76 mol_CO2_/mol_TBAOH_ in isopropyl alcohol (IPA), and 0.86 mol_CO2_/mol_TBAOH_ in EtOH).

**Figure 3:**
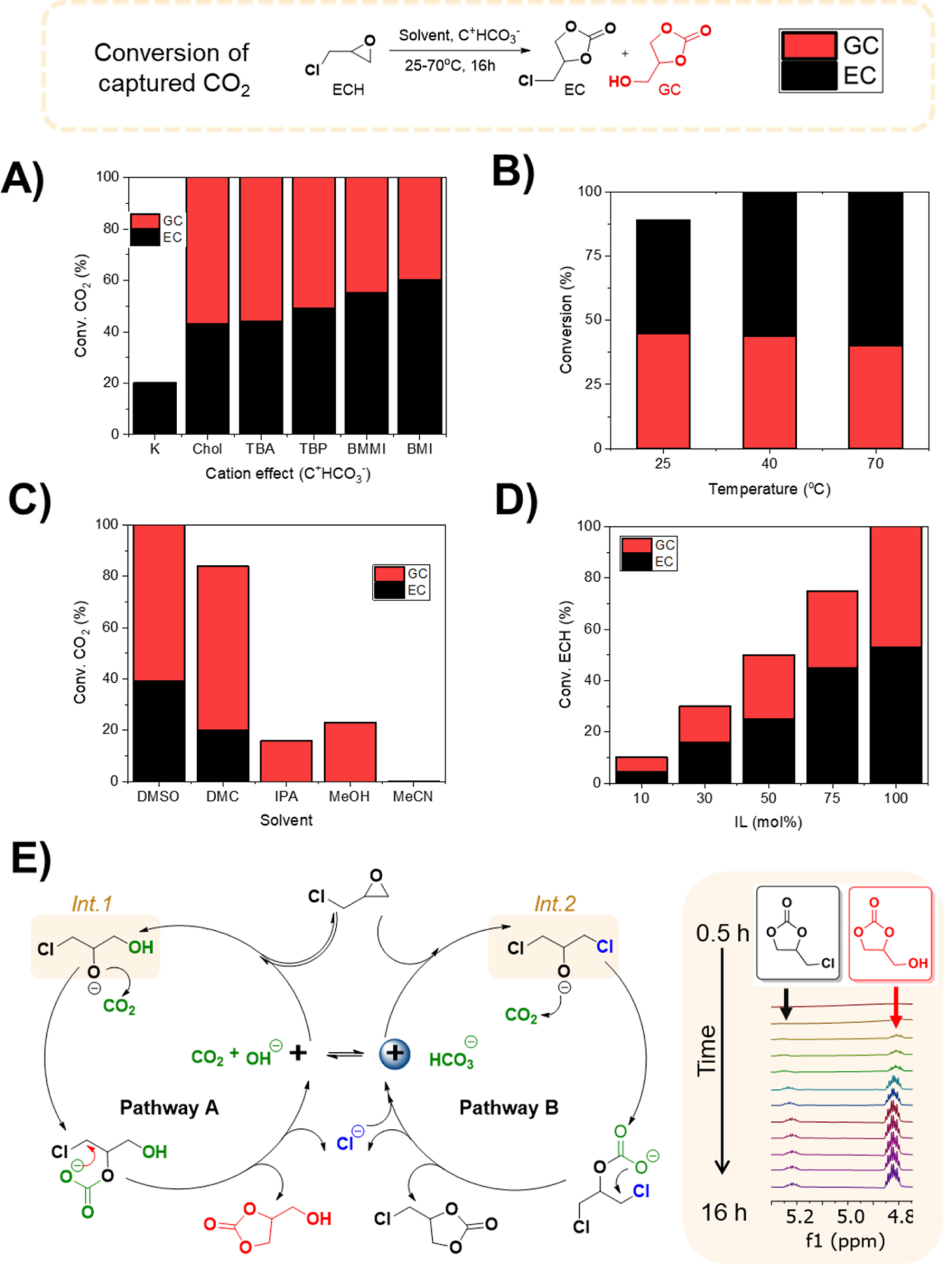
CO_2_ conversion (step 2) evaluation. General reaction conditions: ECH (5 mmol), solvent (0.5 mL), IL (10–100 mol %), 16 h, 25–70 °C. Conversion calculated by ^1^H NMR considering CO_2_ as a limiting reagent. (A) Cation effect (C^+^HCO_3_^–^) (Figures S14–S16); (B) temperature effect (Figure S17); (C) solvent effect (Figure S18); (D) effect of organic salt % (TBA.HCO_3_). (E) Analysis of CO_2_ cycloaddition reaction to ECH using TBA.HCO_3_ in DMSO at 40 °C by ^1^H NMR monitoring and proposed mechanism (Figure S20).

To check the hypothesis, the CO_2_ transformation, step 2 (Figure 1B) was then evaluated. Different IL containing HCO_3_^–^ as an anion (resulting from the previous capture) were used to convert ECH into cyclic carbonates, without employing additional CO_2_ (Figure 3A). In all cases, the CO_2_ source is in lower concentration than the epoxide, thus being the limiting reagent, which implies that conversion and yields should be expressed as a function of the concentration of CO_2_. An equivalent amount of IL and epoxide substrate can be used to obtain complete conversion of the ECH as observed in Figure 3D. All the bicarbonate-based ILs showed complete conversion of CO_2_ at 70 °C (Figure 3A). The use of an inorganic salt (KHCO_3_) showed low conversion (20%) into cyclic carbonate (Figure 3A). Interestingly, two different cyclic carbonates were produced, one containing chlorine, derived from ECH (EC, in black) and another with a hydroxyl group, namely, glycidyl carbonate (GC, in red). The mixture of products is due to the intrinsic reaction mechanism, which will be discussed below.^14^ The complete list of the control experiments can be seen in the Supporting Information (Table S3). From those, it was possible to demonstrate the reaction performance in the absence of co-catalyst and also to further establish the methodology (bubbling CO_2_).

The cation did not significantly influence the total conversion, but some differences in selectivity toward the different carbonates formed were observed. The C2 position of the imidazolium ring did not significantly affect the CO_2_ cycloaddition reaction, thus indicating that, here, the bicarbonate is the active species, and the formation of carbene–CO_2_ adduct is not essential to promote the reaction, as previously reported.^24^ Hence, it is possible to suggest that the HCO_3_^–^ is in equilibrium with OH^–^ and CO_2_. Being able to shift this equilibrium could generate a high concentration of CO_2_ near the catalytic sites, thus favoring the cycloaddition reactions under mild conditions.

The effect of the temperature was also evaluated (Figure 3B). At 25 °C, the reaction is not complete after 16 h (89%). Increasing the temperature to 40 and 70 °C led to complete conversion. Thus, a temperature of 40 °C was selected for further experiments.

After capturing the CO_2_ in different solvents, its conversion was evaluated in MeCN, IPA, MeOH, DMC, and DMSO (Figure 3C). The highest conversion was obtained with DMSO in agreement with that observed for the CO_2_ capture step. The effect of the percentage of organic salt, in this case TBA.HCO_3_ and its concentration were evaluated (Figures 3D and S19). This experiment proves that the reaction is limited by the CO_2_ amount and by having an equivalent quantity of ECH and TBA.HCO_3_, complete conversion can be observed.

To evaluate the reaction mechanism and optimize the selectivity, the reaction was monitored by ^1^H and quantitative ^13^C NMR analyses at 25 °C (Figures 3E and S19). Initially, GC started to form before EC, whereas at the end of the reaction, similar concentration of both products was observed. This suggests two mechanistic pathways (Figure 3E). In pathway A, the HCO_3_^–^ of the IL works as a reservoir of CO_2_ and OH^–^, which works as an IL anion and as a nucleophile to open the epoxide ring. Reversible chemical equilibrium shift driven by small amounts of water leads to the formation of CO_3_^2–^, HCO_3_^–^, and OH^–^, as previously described.^6,12^ At this point, by-products can be formed at higher temperatures, whereas at lower ones, the alkoxide can react with CO_2_ to form the open carbonate intermediate. In this case, the Cl works as leaving group forming the GC. From this point, a new cycle can occur (pathway B) where Cl is participating as the nucleophile, that catalyzes the epoxide opening ring, thus generating the EC in this case. A third route could be possible, where the bicarbonate works as a nucleophile.^25^ However, in this case, another CO_2_ molecule would need to be added to the intermediate to generate the observed products, and the HCO_3_^–^ species would work as the leaving group, thus being a less probable route.

The mechanism proposed was reinforced by testing different epoxide substrates and different CO_2_ sources (Figures S21 and S22). No conversion was observed when epoxy butane was used as the substrate, since there is no leaving group in the molecule. In addition, the test with glycidol demonstrated a low conversion, with the formation of diols as by-products. This confirms that the presence of small amounts of Cl favors the cycloaddition reaction, since Cl is a better nucleophile.

**Figure 4:**
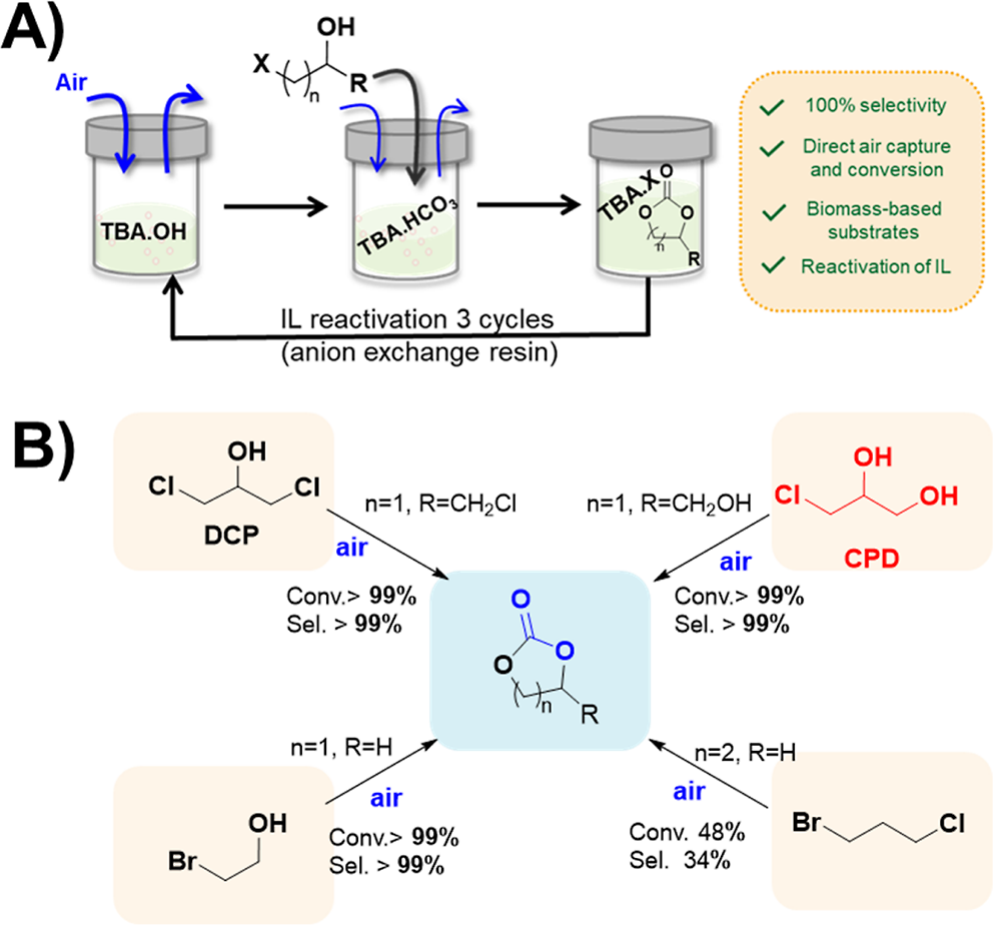
CO_2_ cycloaddition reaction using atmospheric air and different halohydrin substrates. (A) general reaction procedure: ^a^reaction conditions: step 1: TBA.OH (0.5 mmol), DMSO or DMC (0.5 mL), previously bubbled compressed air (16 h, 25 °C); step 2: addition of substrate (0.5 mmol), 16 h, 40 °C, atmospheric air flow (75 mL/min). ^b^Calculated by ^1^H NMR. ^c^Open carbonate species. (B) Scope of reaction.

The reaction mechanism shows that the use of halohydrins (the reaction intermediates int 1 and int 2 shown in Figure 3E) as reagents should lead to selectively produce the different cyclic carbonates. Further advantages are to increase sustainability and reduce the cost of reaction by employing cheap glycerol derivatives [1-chloro-1,2-propanediol (CPD) and 1,3-dichloro-2-propanol (DCP)] (Figure 4A).^26^ Interestingly, full conversion and a complete selectivity toward the respective cyclic carbonates was observed (Figure 4B), thus confirming the reaction mechanism proposed in Figure 3E. Control experiments employing DCP and CPD with TBA.OH without addition of CO_2_ demonstrated the formation of the epoxide (see proposed mechanism, Figure 3E) (Figures S23–S27). This highlights the importance of generating high local concentration of CO_2_ in the reaction media. Other halohydrins were used as substrates and the possibility to produce different five- and six-member cyclic carbonates was proved (see Figures S28–S29).

Finally, the reuse of TBA.OH was demonstrated. An extraction and reactivation protocol was developed to recover the TBA.OH employing a resin exchange column (see Figures S30–S34), since TBA.Cl can be formed during the reaction. The system maintained the activity after at least 3 cycles (Figures S34–S35) using DCP as substrate and DMSO or DMC as solvent.

## Conclusions

We reported a novel DACC methodology capable of completely converting CO_2_ from atmospheric air into cyclic carbonates using epoxides and halohydrins as substrates employing hydroxide-based ILs. Under our experimental conditions, TBA.OH efficiently captured atmospheric CO_2_ (0.98mol_CO2_/mol_IL_) in a variety of solvents and, subsequently, completely converted it into cyclic carbonates. A mechanism of reaction to explain the different products formed has been proposed, where halohydrins were suggested as reaction intermediates. Thus, the reaction employing those halohydrins as substrates was tested with full conversion and 100% of selectivity was observed. Finally, the possibility to recycle the TBA salt was demonstrated. The employment of biomass-based substrates, which are cheaper than ECH; CO_2_ directly sourced from air; and a cheap and commercially available absorbent is highly advantageous and attractive to develop sustainable chemical synthetic routes to generate cyclic carbonates. The simplicity and low cost associated with the capture of CO_2_, combined with the activity demonstrated in the transformation, opens the door to a broad range of DACC methodologies.

## Methodology

### General IL Synthesis Protocols

The following ILs BMI.HCO_3_, BMMI.HCO_3_, TBA.HCO_3_, TBP.HCO_3_, and Chol.HCO_3_ were obtained by anion exchange reaction of the corresponding chloride salt followed by CO_2_ absorption, according to the previous procedure with minor modifications.^27^ A water solution of the chloride salt (10 mmol, 20 mL) was slowly eluted through a column containing 6.00 g of Amberlyst A26 (OH-form), previously prepared with sodium hydroxide 1 M (250 mL). The solution was concentrated and pressurized with 5 bar of CO_2._ The solution was stirred for 4 h at 25 °C, and the solvent was removed under reduced pressure. The final ILs were dried for 16 h, at 60 °C, under vacuum.

### Sorption Experiments

The samples for CO_2_ capture were prepared using a solution of DMSO-*d*_6_ (0.5 mL) and 0.5 mmol of organic salt. The sorption experiments were performed by bubbling the gas (CO_2_ or air) in 5 mm NMR glass tubes with a septum at 25 °C for 15 min when using CO_2_ or 16 h when using air. For the CO_2_ sorption quantification, we have previously established this NMR methodology for CO_2_ quantification in ILs.^22^ Typically, ^13^C NMR inverse gated ^1^H decoupled spectra were acquired using an inversion recovery experiment (zgig) with a relaxation delay of 60 s. The experiments were performed with 64 transients and 64 K data points were collected.

### General Procedure for the Cycloaddition Reaction

Methodology using atmospheric air flow: (step 1) ILs (0.5–1 mmol) and solvent (0.5–1.0 mL) were charged in a glass vial where pre-sorption experiments were performed by bubbling (flow rate 75 mL/min) CO_2_ for 15 min or atmospheric air for 16 h. (Step 2) In the same vial, the correspondent substrate (0.5–5 mmol) was added. The reaction was performed at 40–70 °C for 1–16 h under magnetic stirring and atmospheric air flow. The product was analyzed by ^1^H NMR spectroscopy to determine the conversion and selectivity of cyclic carbonates.

### Reactivation of TBA.OH for Recycle Tests

The ILs were recycled by washing the reaction mixture with diethyl ether (3 × 10 mL) and extraction with cold CHCl_3_ (3 × 10 mL) and water. The aqueous phase was used to reactivate the ILs, and the organic phase was used to separate the product. The reactivation of ILs was performed using an ion exchange resin (Amberlyst A26) (OH-form), according to the procedure described in the synthesis of ILs..

## Supporting Information Available

NMR analysis of ionic liquid and CO_2_ capture and CO_2_ conversion experiments. Quantification of sorption capacity and conversion and recycle experiments (PDF)
