# Supplementary material for: Direct Air Capture and Integrated Conversion of Carbon Dioxide into Cyclic Carbonates with Basic Organic Salts
Source: ACS Sustain Chem Eng. 2023 Jun 23;11(26):9613–9. doi: 10.1021/acssuschemeng.3c00890 (PMC10324388; doi:10.1021/acssuschemeng.3c00890)
Supplement: Supplementary file 1 — sc3c00890_si_001.pdf [file sc3c00890_si_001.pdf]

**Supporting information for**

# Direct air capture and integrated conversion of carbon dioxide into cyclic carbonates with basic organic salts

Marcileia Zanatta,*[a] Eduardo García-Verdugo,[b] Victor Sans,*[a]

[a] Institute of Advanced Materials (INAM), Univesitat Jaume I, Avda Sos Baynat s/n, 12071, Castellón, Spain
[b] Departamento de Química Inorgánica y Orgánica, Grupo de Química Sostenible y Supramolecular Universidad Jaume I, E-12071 Castellón, Spain

*sans@uji.es*

*zanatta@uji.es*

This document presents 27 pages with 37 figures and 3 tables.

**Table of contents**

1. Experimental details 2
  1.1. Materials and Reagents 2
  1.2. Equipment and analyses 2
  1.3. Ionic liquid and cyclic carbonate characterization 2
2. $CO_2$ capture experiments (step 1) 6
3. $CO_2$ conversion using concentrated source (step 2) 10
  3.1. Control experiments 10
  3.2. IL screening and conversion mechanism evaluation 13
4. DACC experiments 18
  4.1. Substrate evaluation 18
  4.2. Recycle experiments 22
5. References 26

# 1. Experimental details

## 1.1. Materials and Reagents

All reagents and solvents used were commercially available: styrene oxide (97%, Sigma Aldrich), epichlorohydrin (98%, Sigma Aldrich), tetrabutylammonium hydroxide (1 mol.$L^{-1}$ in methanol, Sigma Aldrich), tetrabutylammonium bromide (98%, Sigma Aldrich), tetrabutylphosphonium hydroxide (40% in water, TCI), 1-$^{n}$butyl-3-methylimidazolium chloride (98%, Sigma Aldrich); 1,2-dimethylimidazole (98%, IoLiTec); chlorobutane (99% TCI), 3-chloro-1,2-propanediol (98%, Sigma Aldrich) and 1,3-dichloro-2-propanol (98%, Sigma Aldrich), acetic acid (99.7%, Sigma Aldrich), DMSO-$d_6$ (Eurisotop 99.8%), Amberlyst A26 (OH-form). All the solvents were used as received from VWR Chemicals. Carbon dioxide was supplied by Air Liquide with purity above 99.998 % and atmospheric air containing 0.04% of $CO_2$.

## 1.2. Equipment and analyses

$^{1}$H-NMR, $^{13}$C-{$^{1}$H} NMR experiments were carried out using a Bruker AVANCE III HD 400 spectrometer (300 or 400 MHz for 1H), with BBFO 5mm probe.
*$^{13}$C NMR quantification***:** Quantitative $^{13}$C NMR spectra can be obtained using the inverse gated $^{1}$H decoupled experiment with a correct relaxation delay that ensures full relaxation of the $^{13}$C nuclei, as have been demonstrated previously by us for $CO_2$ quantification.[1] $^{13}$C NMR inverse gated $^{1}$H decoupled spectra were acquired using an inversion recovery experiment (*zgig*) with a relaxation delay of 60 seconds, set to 5*$T_1$ according to the previously determined $CO_2$ $T_1$. Typically, in each experiment 128 transients with 64K data points were collected corresponding to an average duration of 2 h.

## 1.3. Ionic liquid and cyclic carbonate characterization

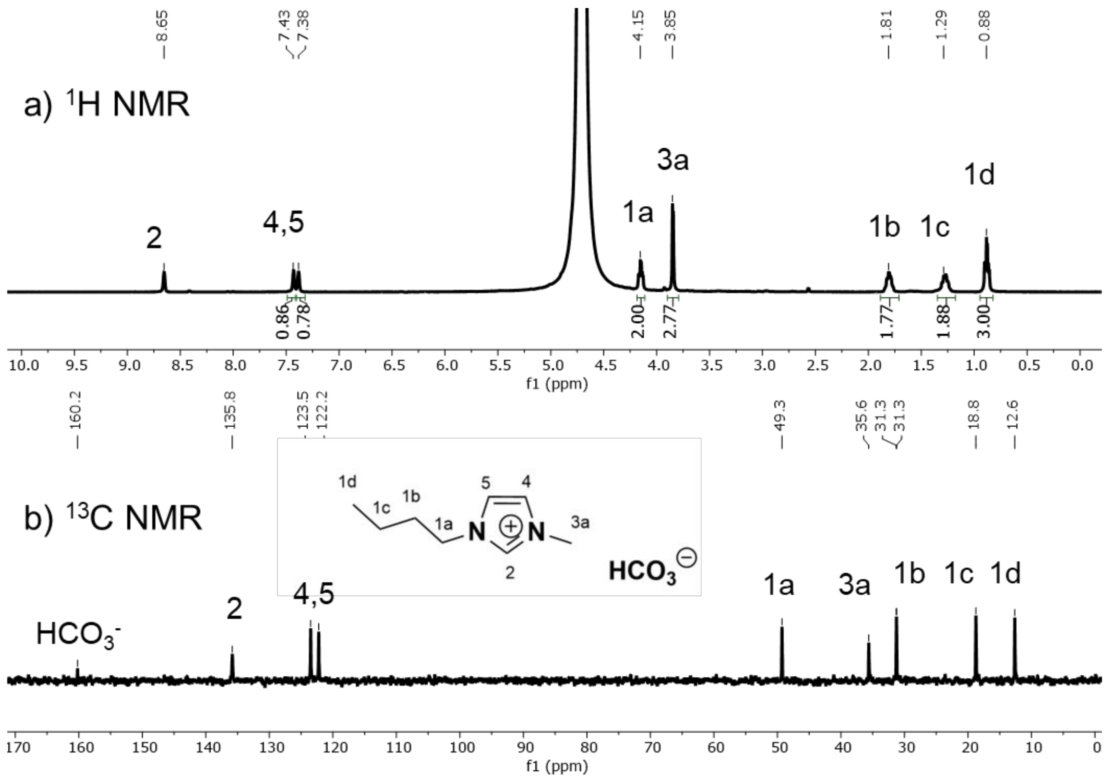


Figure S 1. NMR spectra (400 MHz, 298 K, $D_2O$) of BMI·$HCO_3$ (a) $^1$H NMR; (a) $^{13}$C NMR.

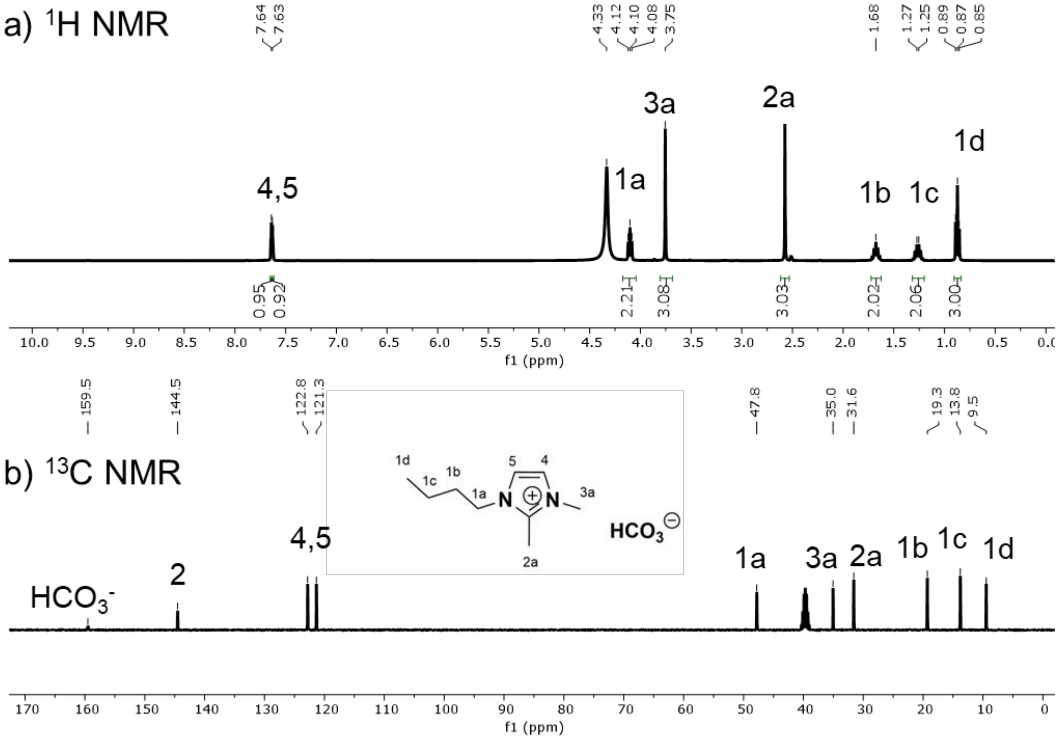


Figure S 2. NMR spectra (400 MHz, 298 K, DMSO-$d_6$) of BMMI·$HCO_3$ (a) $^1$H NMR; (a) $^{13}$C NMR.

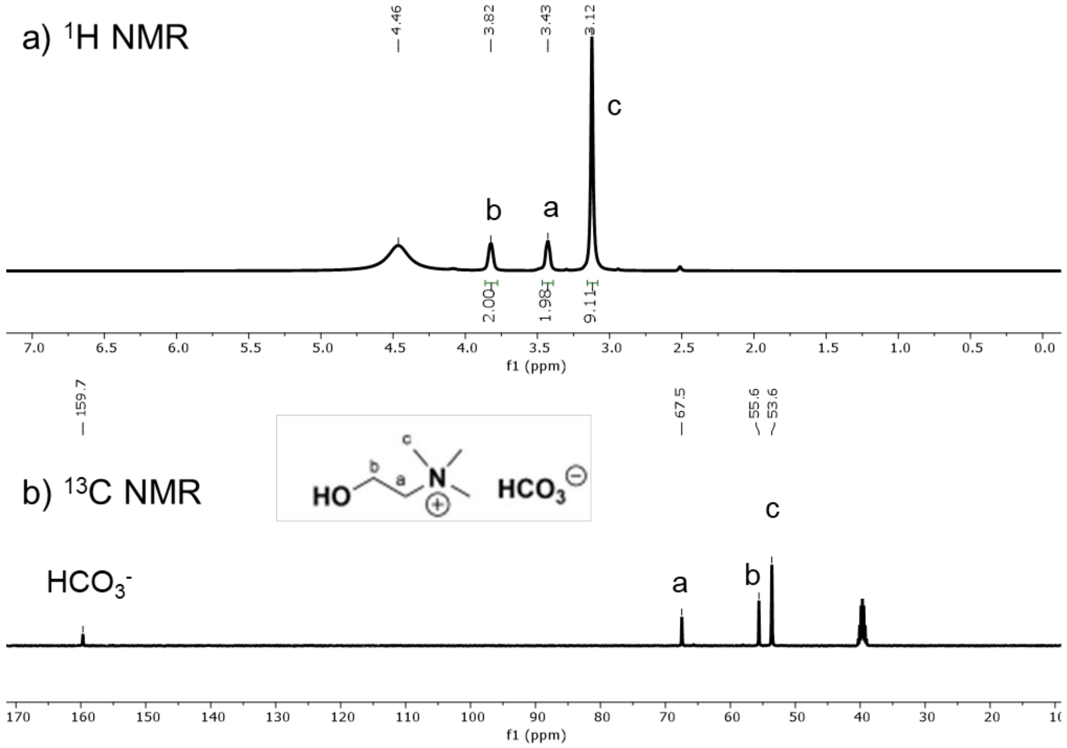


Figure S 3. NMR spectra (400 MHz, 298 K, DMSO-$d_6$) of Chol·$HCO_3$ (a) $^1$H NMR; (a) $^{13}$C NMR.

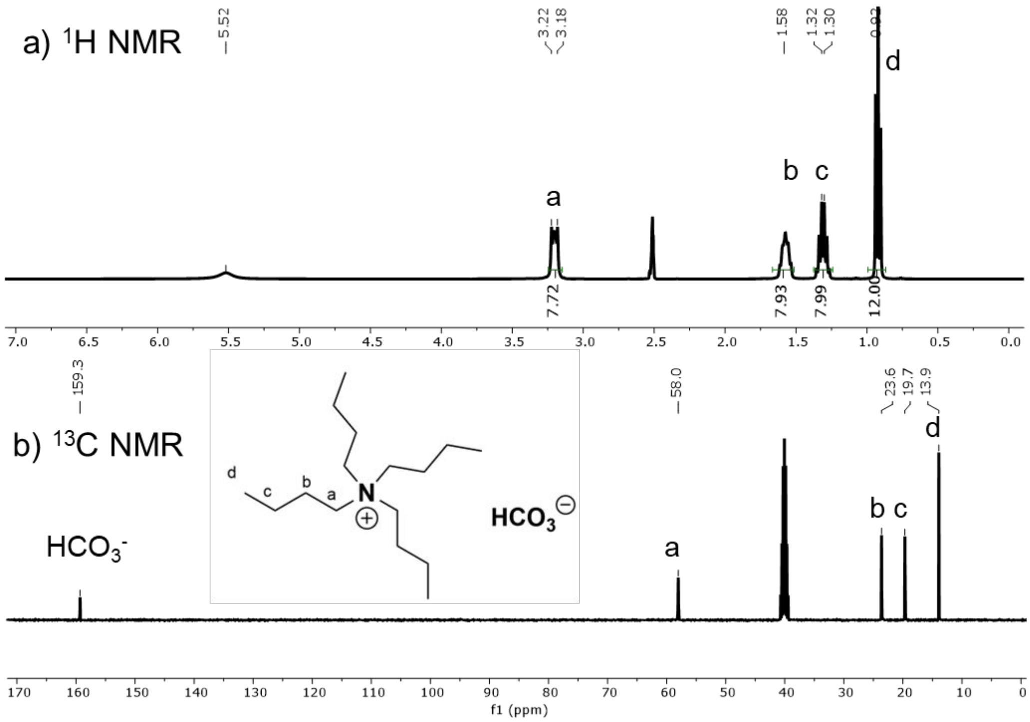


Figure S 4. NMR spectra (400 MHz, 298 K, DMSO-$d_6$) of TBA·$HCO_3$ (a) $^1$H NMR; (a) $^{13}$C NMR.

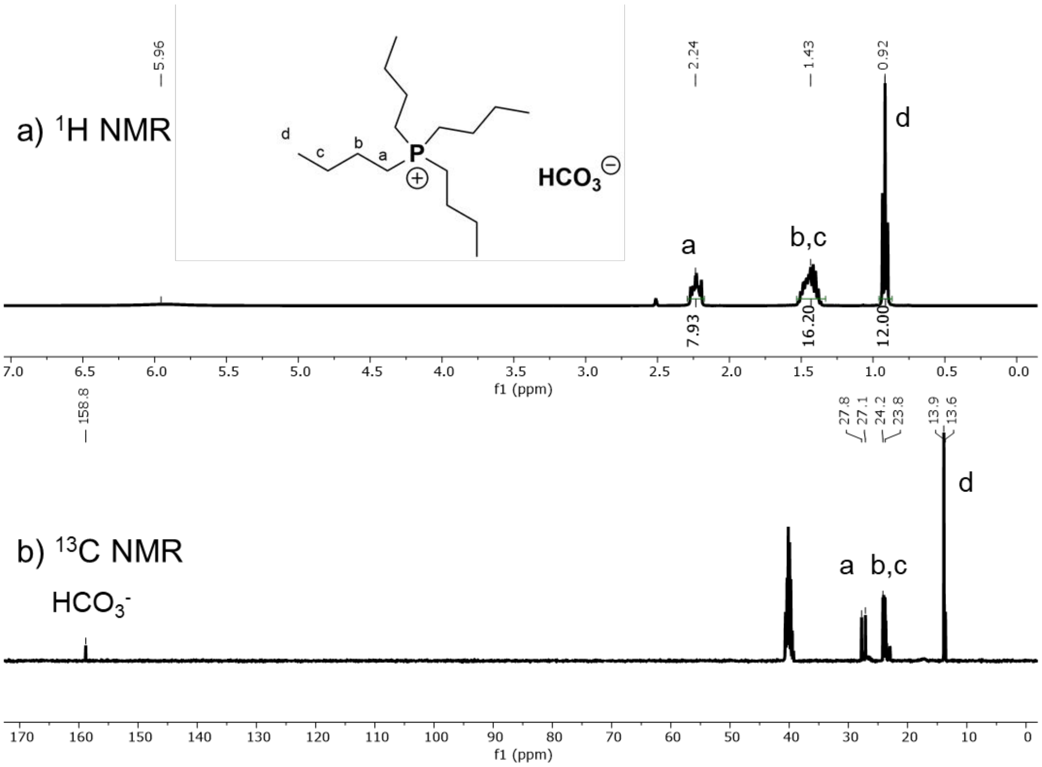


Figure S 5. NMR spectra (400 MHz, 298 K, DMSO-$d_6$) of TBP·$HCO_3$ (a) $^1$H NMR; (a) $^{13}$C NMR.

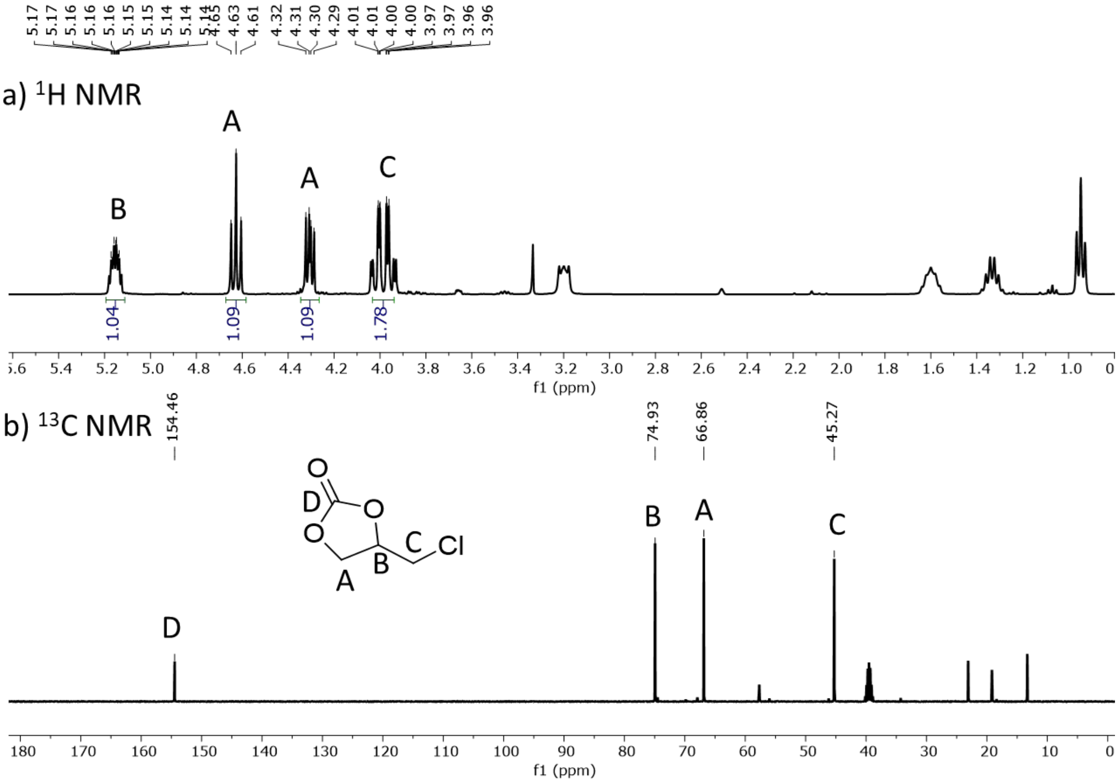


Figure S 6. {$^1$H} NMR spectra (400 MHz, 298 K) of **cyclic carbonate from epichlorohydrin**. Reaction conditions: ECH (5 mmol), TBAB (10 mol%), 4 h, 70 $^o$C, 5 bar $CO_2$. (a) $^1$H NMR; (b) $^{13}$C NMR.

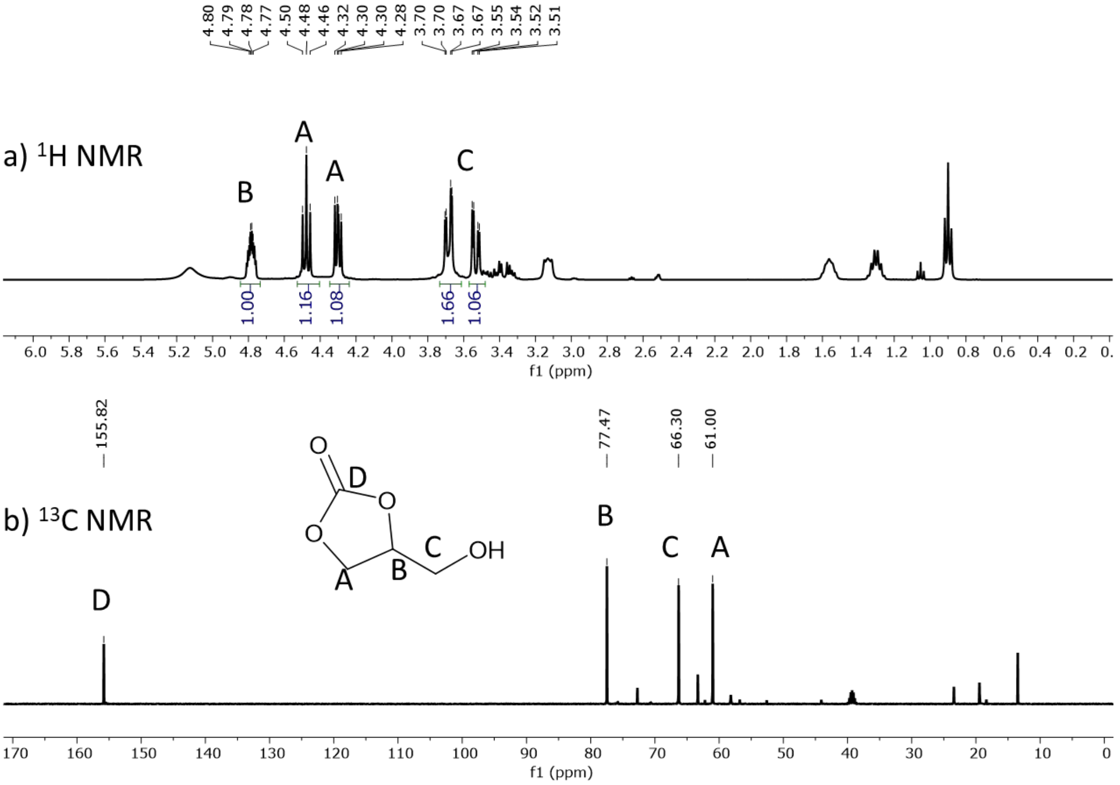


Figure S 7.{NMR spectra (400 MHz, 298 K) of **cyclic carbonate from glycidol**. Reaction conditions: glycidol (5 mmol), TBAB (10 mol%), 4 h, 70 °C, 5 bar $CO_2$. (a) $^{1}H$ NMR; (b) $^{13}C$ NMR.

## 2. $CO_2$ capture experiments (step 1)

**Sorption experiments:** The samples for $CO_2$ capture were prepared using a mixture of solvents DMSO-$d_6$ (0.5 mL) and correspond amount of organic salts (0.5 – 1.5 mmols). All the sorption experiments were performed by bubbling the gas ($CO_2$ or air) in 5 mm NMR glass tubes with a septum at room temperature for 15 min ($CO_2$) or 16h (air). For the $CO_2$ sorption quantification, we have previously established this NMR methodology for $CO_2$ quantification in ILs.

First of all the effect of cation was evaluated (Table S1) and secondly the effect of solvent (Table S2).

**Table S1.** $CO_2$ sorption after bubbling gas from different sources in different IL solutions.

| | | $CO_2$ [a] | Air [b] |
|---|---|---|---|
| Entry | $C^+OH$ | $mol_{CO2tot}/mol_{IL}$ [c] | $mol_{CO2tot}/_{IL}$ [c] |
| 1 | BMI | - [d] | - [d] |
| 2 | BMMI | - [d] | - [d] |
| 3 | TBP | 0.16 | 0 |
| 4 | Chol | 0.51 | 0 |
| 5 | TBA | 0.95 | 0.98 |

*Sorption conditions:* bubbling gas at 25°C in IL solution (1 mol $L^{-1}$ in DMSO). [a] 15 min $CO_2$. [b] 16 h of atmospheric air. [c] Calculated by quantitative $^{13}C$ NMR (Figures S17-S18). [d] Degradation of IL.

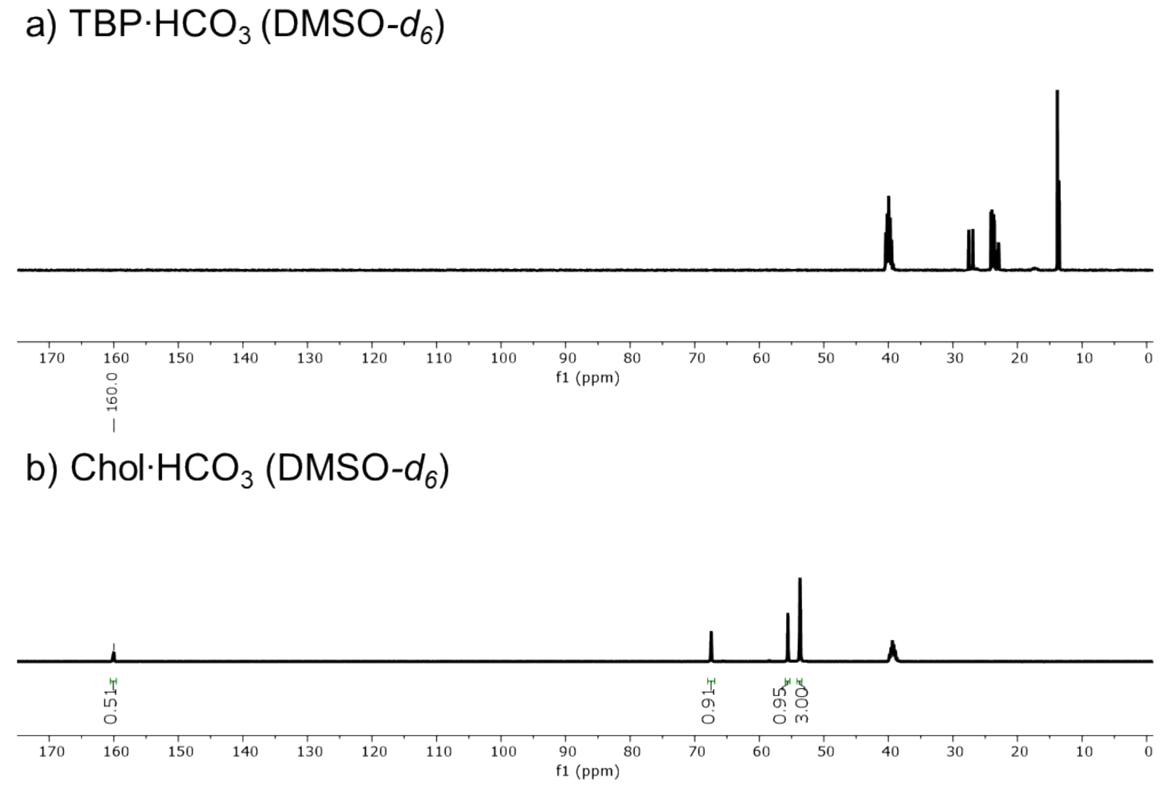


Figure S 8 $^{13}C$-{$^{1}H$} NMR spectra (100 MHz, 298 K) of IL (1 mol.L$^{-1}$ DMSO-*d*$_6$) after 15 min of bubbling $CO_2$: a) TBP.OH*;* b) Chol.OH

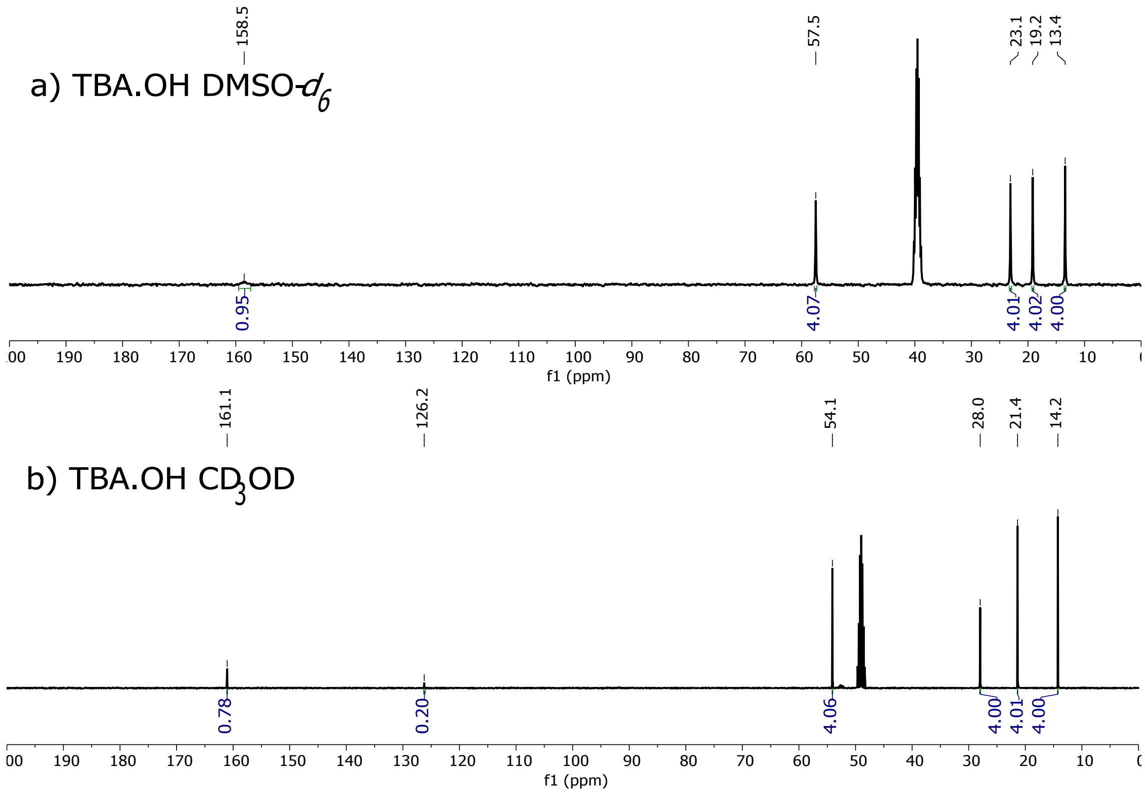


Figure S 9 $^{13}C$-{$^{1}H$} NMR spectra (100 MHz, 298 K) of TBA.OH (1 mol.L$^{-1}$) after 15 min of bubbling $CO_2$: a) DMSO-*d*$_6$*;* b) $CD_3OD$.

**Table S2.** $CO_2$ sorption capacity of TBA.OH in different solvents after bubbling atmospheric air.[a]

| Entry | Solvent | $mol_{CO2tot}/mol_{IL}$ |
|---|---|---|
| 1 | DMSO-$d_6$ | 0.98 |
| 2 | $CD_3OD$[d] | 0.21 |
| 3 | EtOH[b] | 0.54 |
| 4 | IPA[b] | 0.76 |
| 5 | DMC[b] | 0.95 |
| 6 | $D_2O$ | 0.86 |

[a]*Sorption conditions*: 16 h of atmospheric air flow at 25°C in TBA.OH solution (1 mol $L^{-1}$). [b] Partial solvent evaporation. Constant addition of solvent was employed to maintain constant the sorption system.

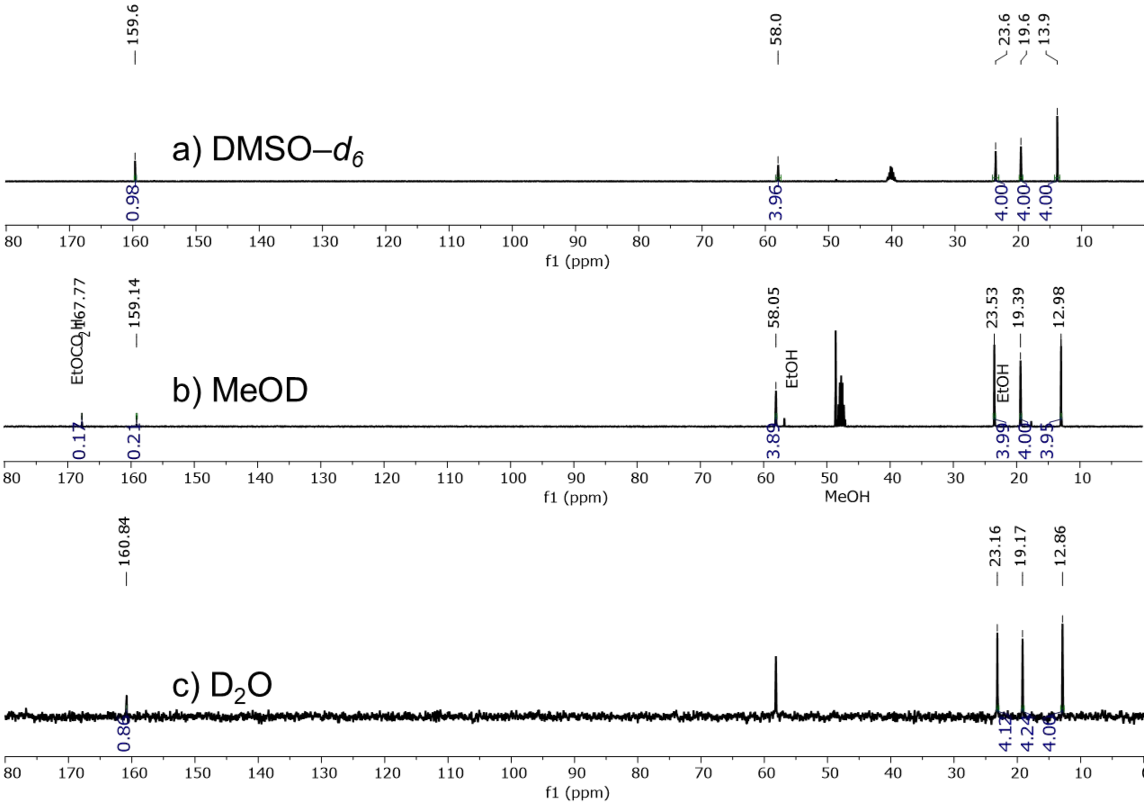


Figure S 10.$^{13}$C-{$^1$H} NMR spectra (100 MHz, 298 K) of TBA.OH (1 mol.$L^{-1}$) after 16 h of bubbling air: a) DMSO-$d_6$; b) $CD_3OD$; c) $D_2O$.

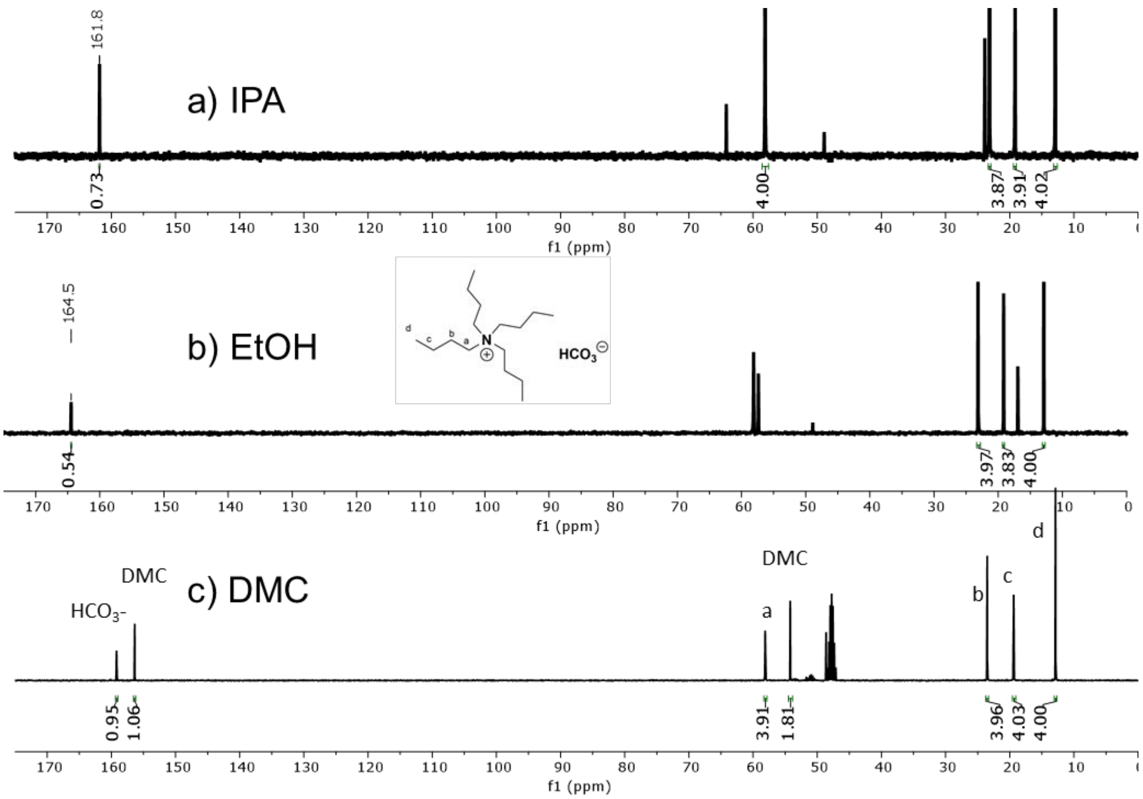


Figure S 11.$^{13}$C-{$^{1}$H} NMR spectra (100 MHz, 298 K) of TBA.OH (1 mol.L$^{-1}$) after 16 h of bubbling air: a) IPA; b) EtOH; c) DMC.

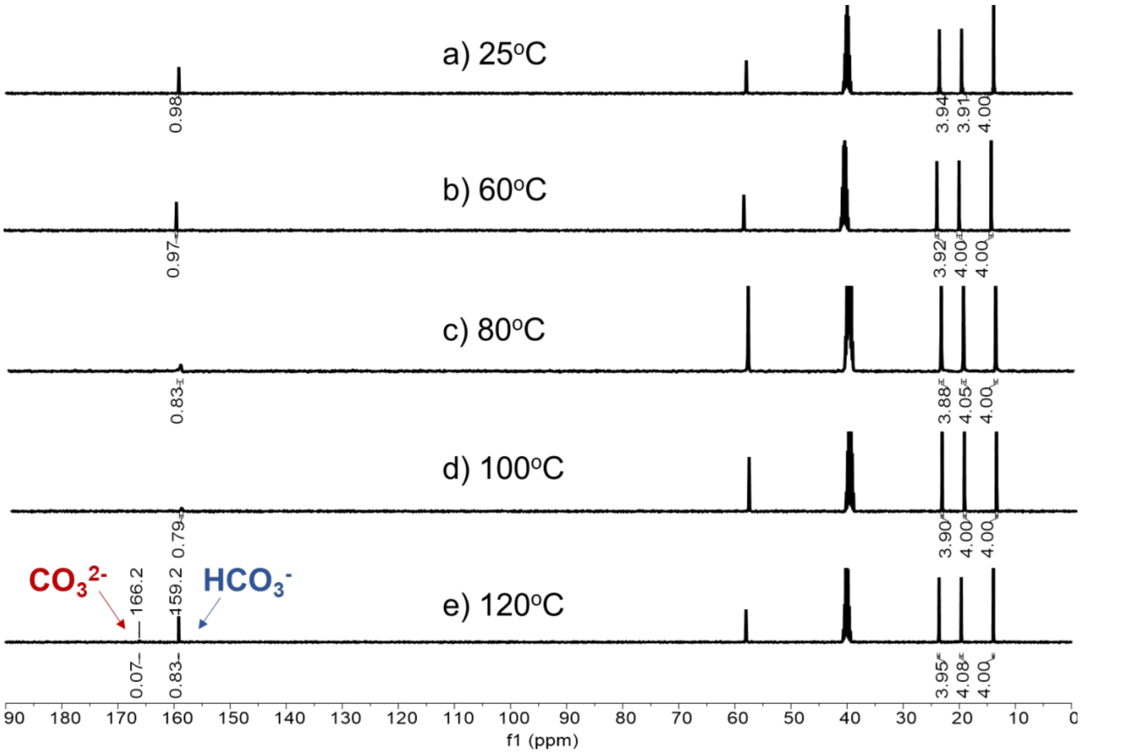


Figure S 12.$^{13}$C-{$^{1}$H} NMR spectra (100 MHz, 298 K) of desorption experiments using TBA.$HCO_3$ (1 mol.L$^{-1}$ in DMSO-$d_6$).

Once captured by forming $HCO_3^-$, the $CO_2$ can't be desorbed even heating at 120°C. However, the bicarbonate can be use as $CO_2$ source in order to perform cycloaddition reaction as observed in the subsequential section (section 3).

## 3. $CO_2$ conversion using concentrated source (step 2)

### 3.1. Control experiments

**General procedure for the cycloaddition reaction:**

*Methodology using balloon (method 1):* ILs (5-30 mol%), solvent (0.5 mL) and the correspondent epoxide (5 mmol) were charged in glass vial connected to a $CO_2$ balloon. The reaction was performed at 40 °C - 70 °C under 1-16 h under magnetic stirring. The product was analysed by $^1$H NMR spectroscopy to determine the conversion and selectivity of cyclic carbonates.

*Methodology using gas flow (method 2): Step 1)* ILs (0.5 - 1 mmol) and solvent (0.5 – 1.0 mL) were charged in glass vial where pre-sorption experiments were performed by bubbling (flow rate 75 mL/min) $CO_2$ for 15 min or atmospheric air for 16 h. *Step 2)* In the same vial the correspondent substrate (0.5 - 5 mmol) was added. The vials were closed with septum and reaction were performed at 40-70 °C under 1-16 h under magnetic stirring. The product was analysed by $^1$H NMR spectroscopy to determine the conversion and selectivity of cyclic carbonates.

Control experiments using balloon of $CO_2$ were performed to comparative purpose. [2-12] The conversion using $CO_2$ balloon (method 1) was investigate for BMI.$HCO_3$ and TBAB as catalyst (Table S2, entries 1-4). High conversions (75-99%) of $CO_2$ into cyclic carbonate was observed. Using the method 2, even with previous bubbling $CO_2$, no conversion can be observed using just TBAB as catalyst (Table S1 entries 4-5), since the bromide IL are not good $CO_2$ sorbents.

In order to evaluate the influence of $CO_2$ amount available, different concentrations of BMI.$HCO_3$ were used to the $CO_2$ cycloaddition reaction (entries 7-9). The $CO_2$ amount was confirmed by $^{13}$C quantitative NMR (Figure S12), as expected IL containing $HCO_3^-$ as anion present 1mol $CO_2$/molIL. Considering the $CO_2$ as the limiting reactant, maximum conversion was observed for all the experiments, confirming the $CO_2$ mass balance. The increases in the IL concentration from 10 to 30 mol%, reduce the conversion into EC and increase the formation of glycidol carbonate (GC).

**Table S3.** Control experiments of reaction between ECH and $CO_2$.[a]

| Entry | IL. | IL (mol%) | Cocat. | Solv. | Method X | Conv ECH (%)[b] | Conv $CO_2$. (%)[c] | EC (%) | GC (%) |
|---|---|---|---|---|---|---|---|---|---|
| **1** | - | - | TBAB | DMSO | 1 | 75 | - | 100 | - |
| **2** | BMI.$HCO_3$ | 10 | TBAB | DMSO | 1 | > 99 | - | 91 | 9 |
| **3** | BMI.$HCO_3$ | 10 | - | DMSO | 1 | 80 | - | 65 | 35 |
| **4** | - | - | TBAB | DMSO | 2 | < 2 | - | 100 | - |
| **5** | - | - | TBAB | - | 2 | < 1 | - | 100 | - |
| **6** | BMI.$HCO_3$ | 20 | - | DMSO | 2 | 20 | > 99 | 60 | 40 |
| **7** | BMI. $HCO_3$ | 10 | TBAB | DMSO | 2 | 9 | > 99 | 61 | 39 |
| **8** | BMI. $HCO_3$ | 20 | TBAB | DMSO | 2 | 20 | > 99 | 56 | 44 |
| **9** | BMI. $HCO_3$ | 30 | TBAB | DMSO | 2 | 28 | > 99 | 40 | 60 |

[a] *Reaction conditions*: ECH (5 mmol), DMSO (0.5 mL), IL (10-30 mol%), TBAB (10 mol%), 16 h, 70 °C. [b] Calculated by $^1$H NMR using ECH signals as internal standard. [c] Calculated by $^1$H NMR using ILs signals as internal standard (considering 1 mol$CO_2$/mol IL) (Figures S8-S10). Method I: *$CO_2$ balloon*

during the reaction with ECH. Method II*: $CO_2$ previous bubbled*, followed by removed the $CO_2$ source and addition of ECH.

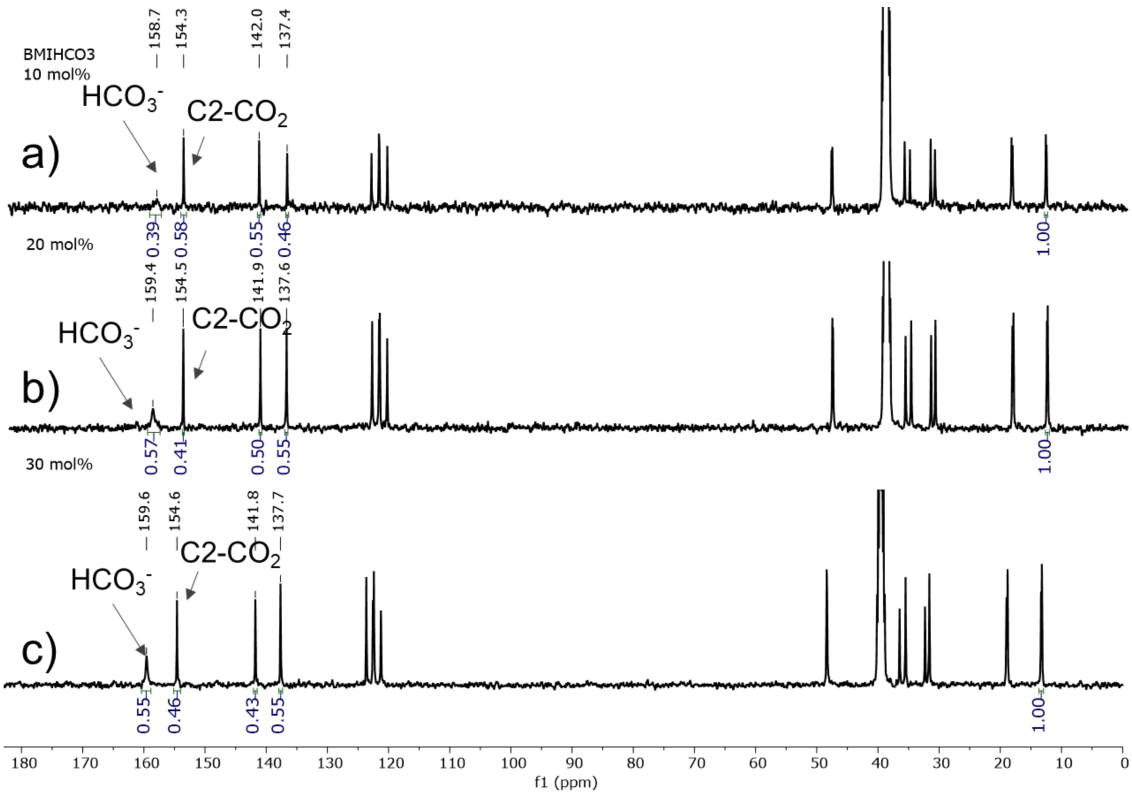


Figure S 13. {$^{13}$C-{$^{1}$H} NMR spectra (100 MHz, 298 K) of BMI·$HCO_3$ after 15 min of bubbling $CO_2$: (a) 1 mol.L$^{-1}$ (same of 10 mol% in catalytic test); (b) 2 mol.L$^{-1}$ (same of 20 mol% in catalytic test) (c) 3 mol.L$^{-1}$ (same of 30 mol% in catalytic test).

## 3.2. IL screening and conversion mechanism evaluation

Test using different bicarbonate-based salts to convert $CO_2$ into cyclic carbonate using epoxide as substrate without the addition of $CO_2$ was performed as a proof-of-concept.

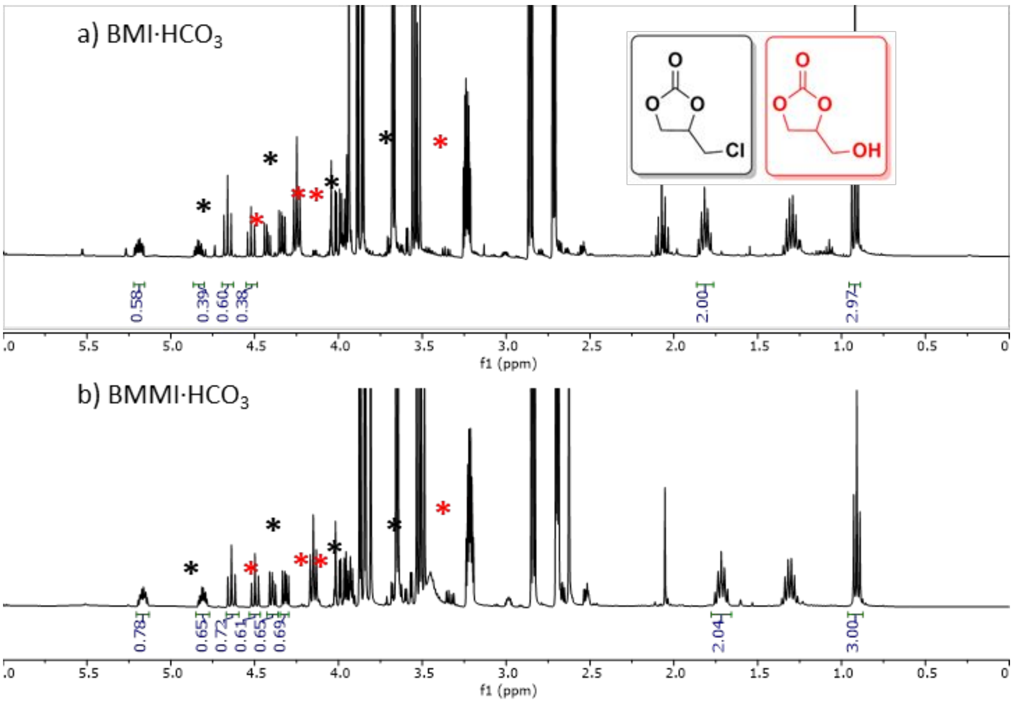


Figure S 14. {$^{1}$H} NMR spectra (400 MHz, 298 K) of cycloaddition reaction, ECH (5 mmol), IL (10 mol%), 16 h, 70 $^{o}C$, $CO_2$ previously bubbled, 0.5 mL DMSO. (a) BMI·$HCO_3$; (b) BMMI·$HCO_3$.

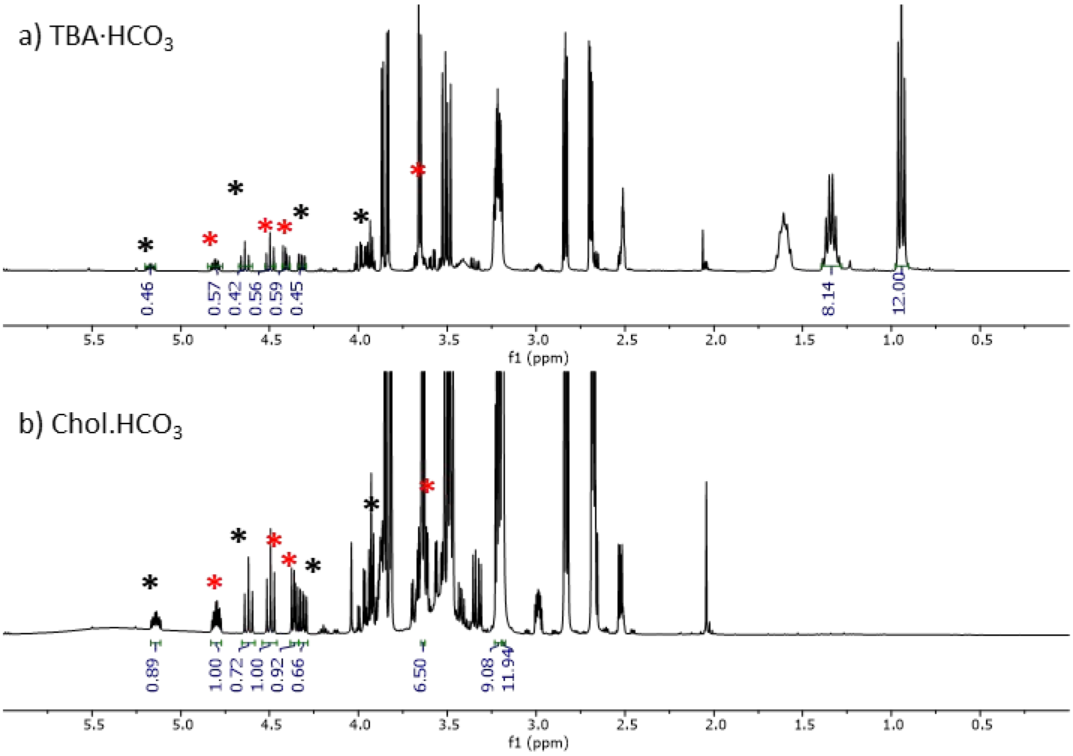

Figure S 15. {$^{1}$H} NMR spectra (400 MHz, 298 K) of cycloaddition reaction, ECH (5 mmol), IL (10 mol%), 16 h, 70 °C, $CO_2$ previously bubbled, 0.5 mL DMSO. (a) TBA·$HCO_3$; (b) Chol.$HCO_3$.

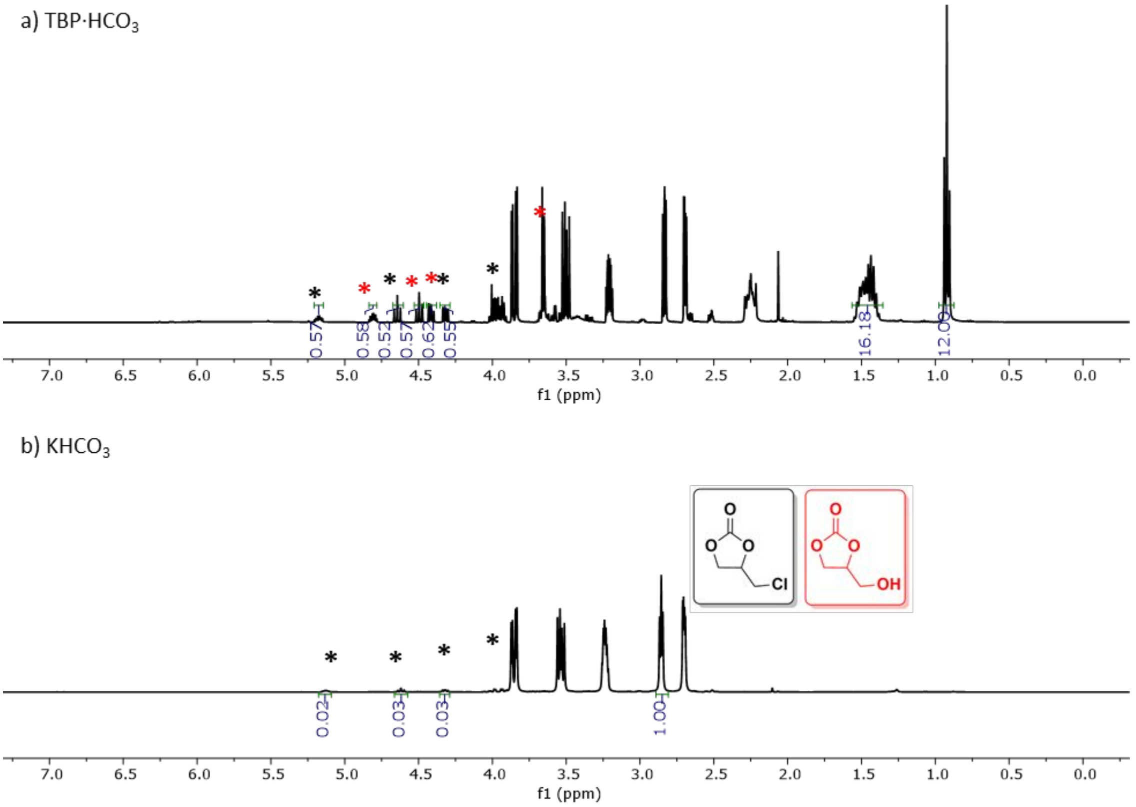


Figure S 16. {$^{1}$H} NMR spectra (400 MHz, 298 K) of cycloaddition reaction, ECH (5 mmol), $KHCO_3$. IL (10 mol%), 16 h, 70 °C, $CO_2$ previously bubbled, 0.5 mL DMSO. (a) TBP·$HCO_3$; (b) $KHCO_3$.

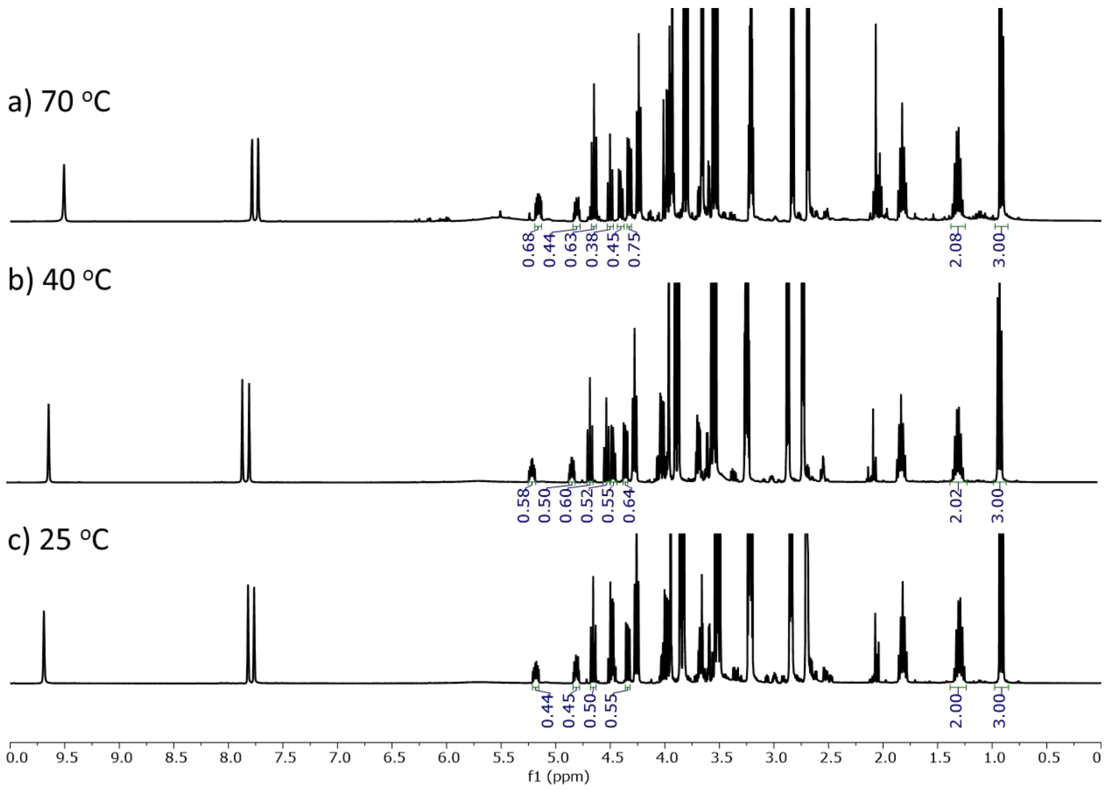


Figure S 17. {$^{1}$H} NMR spectra (400 MHz, 298 K) of cycloaddition reaction, ECH (5 mmol), $BMI.HCO_3$ (10 mol%), 16 h, 0.5 mL DMSO. (a) 70 °C; (b) 40 °C; (c) 25 °C.

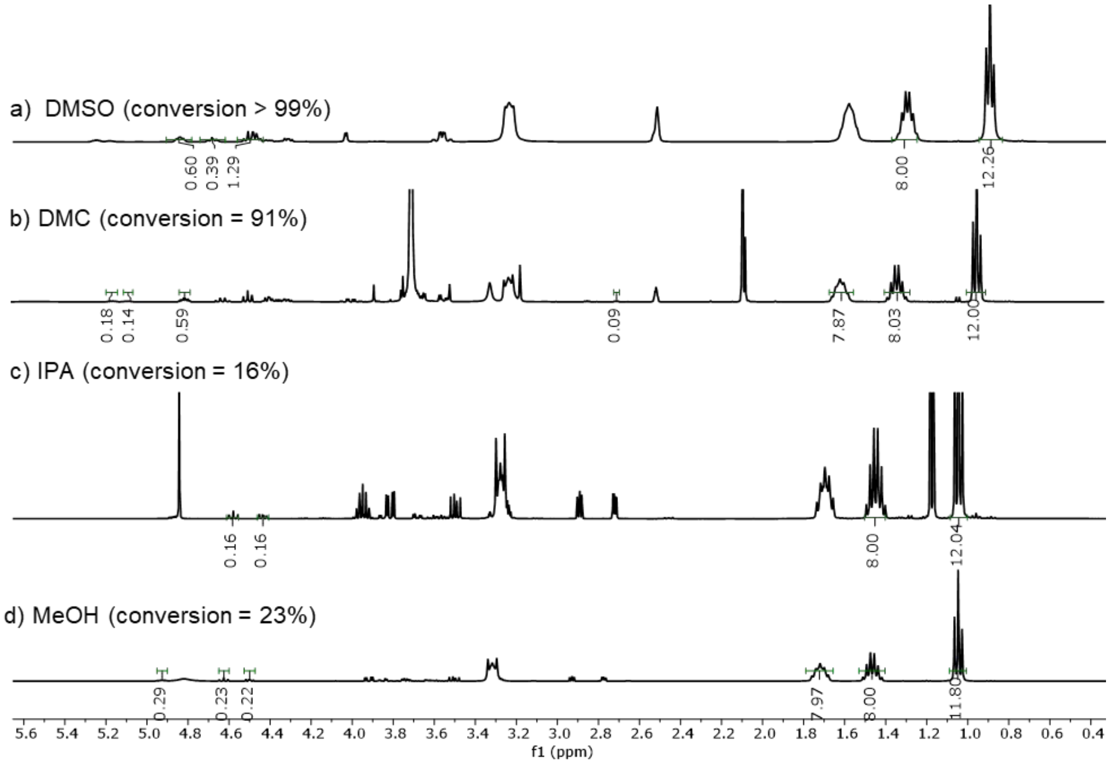


Figure S 18. $^1H$ NMR spectra (400 MHz, 298 K) of cycloaddition reaction, ECH, $TBA.HCO_3$ (100 mol%) atmospheric $CO_2$ continuous flow, 0.5 mL solvent. a) DMSO-$d_6$; b) dimethylcarbonate ; c) IPA; d) $CD_3OD$

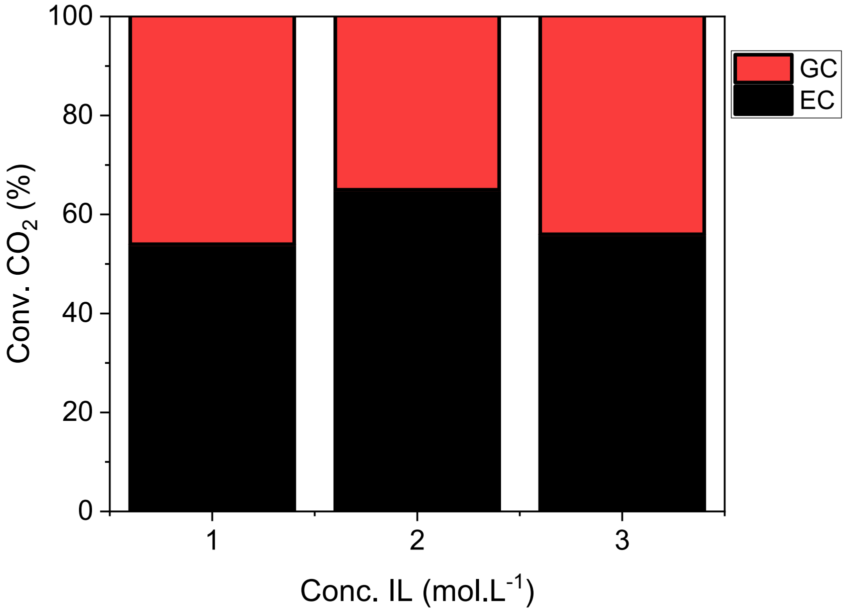


Figure S 19. Effect of IL concentration in $CO_2$ conversion to epoxide using ECH as substrate. TBA.$HCO_3$ variation related to DMSO (solution concentration).

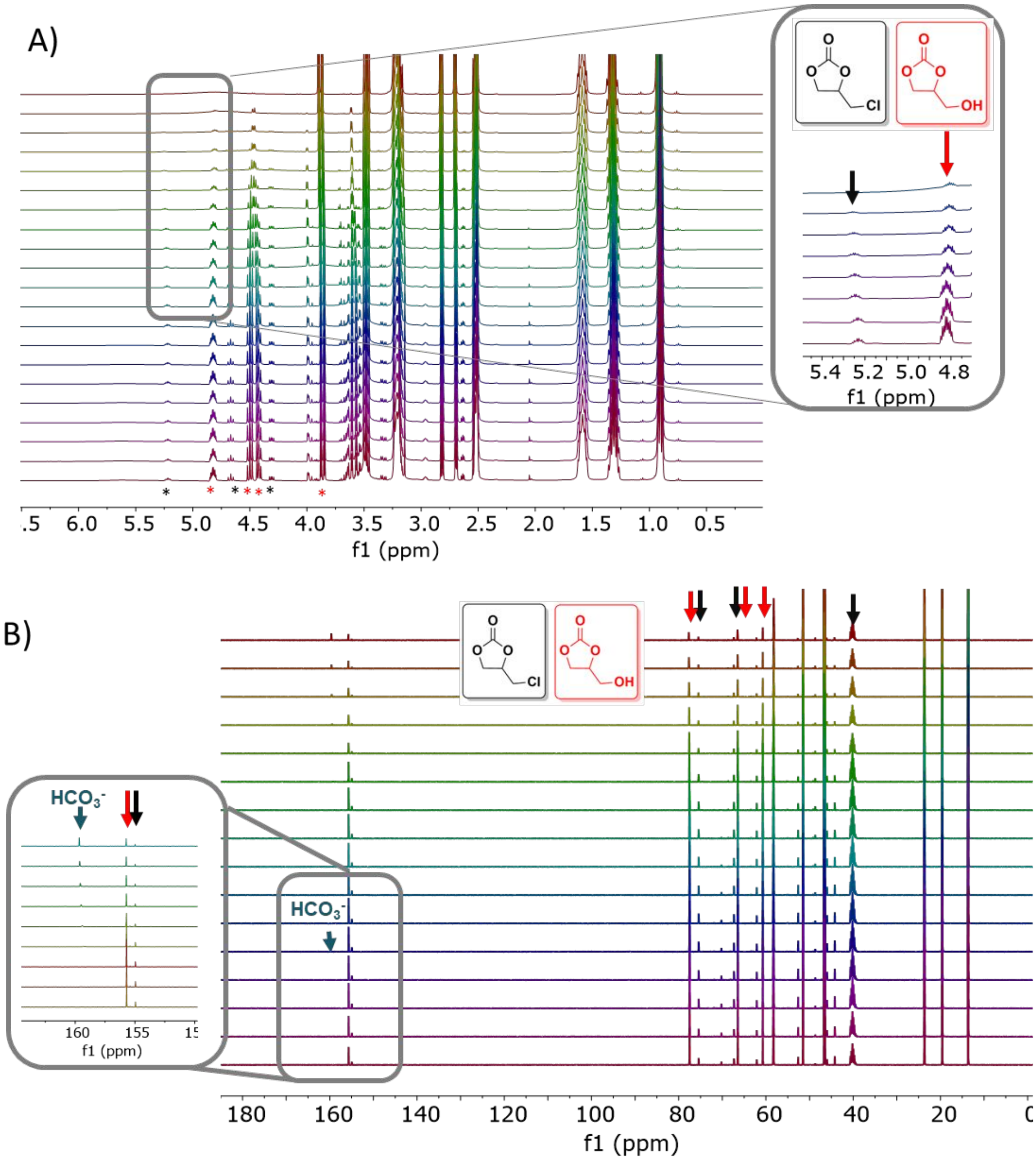


Figure S 20. NMR monitoring analysis of $CO_2$ cycloaddition reaction to ECH using TBA.$HCO_3$. *Reaction conditions*: ECH (5 mmol), TBA.$HCO_3$ (10 mol%), DMSO (0.5 mL), 25ºC, 16 h, previously bubbled $CO_2$. A) $^1$H NMR, B) $^{13}$C NMR.

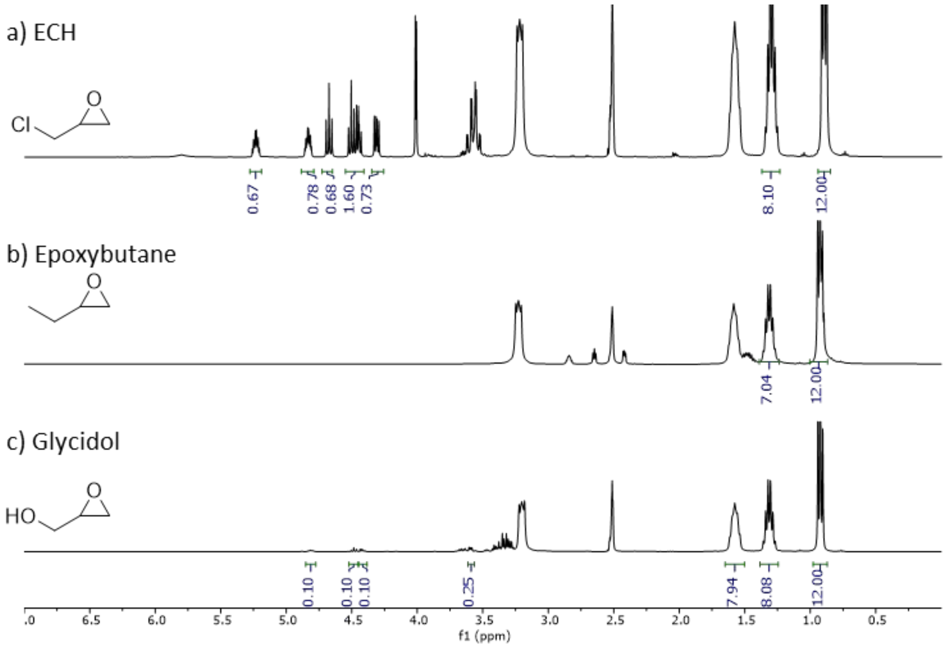


Figure S 21. $^{1}$H NMR spectra (400 MHz, 298 K) of cycloaddition reaction, substrate, TBA.$HCO_3$ (Eq. molar), $CO_2$ balloon, 0.5 mL DMSO, 40 °C, 16h. (a) ECH; (b) epoxybutane; (c) glycidol.

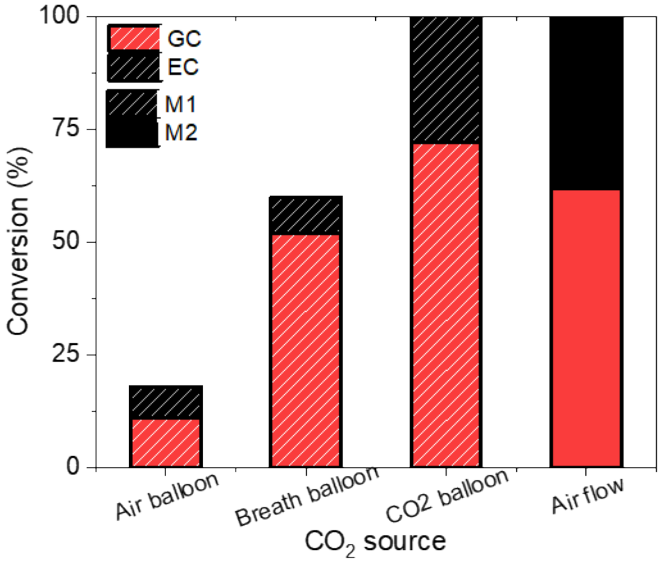


Figure S 22. Cycloaddition reaction using different $CO_2$ sources: ECH, TBA.$HCO_3$ (Eq. molar), 0.5 mL DMSO, 40 °C, 16h.

## 4. DACC experiments

### 4.1. Substrate evaluation

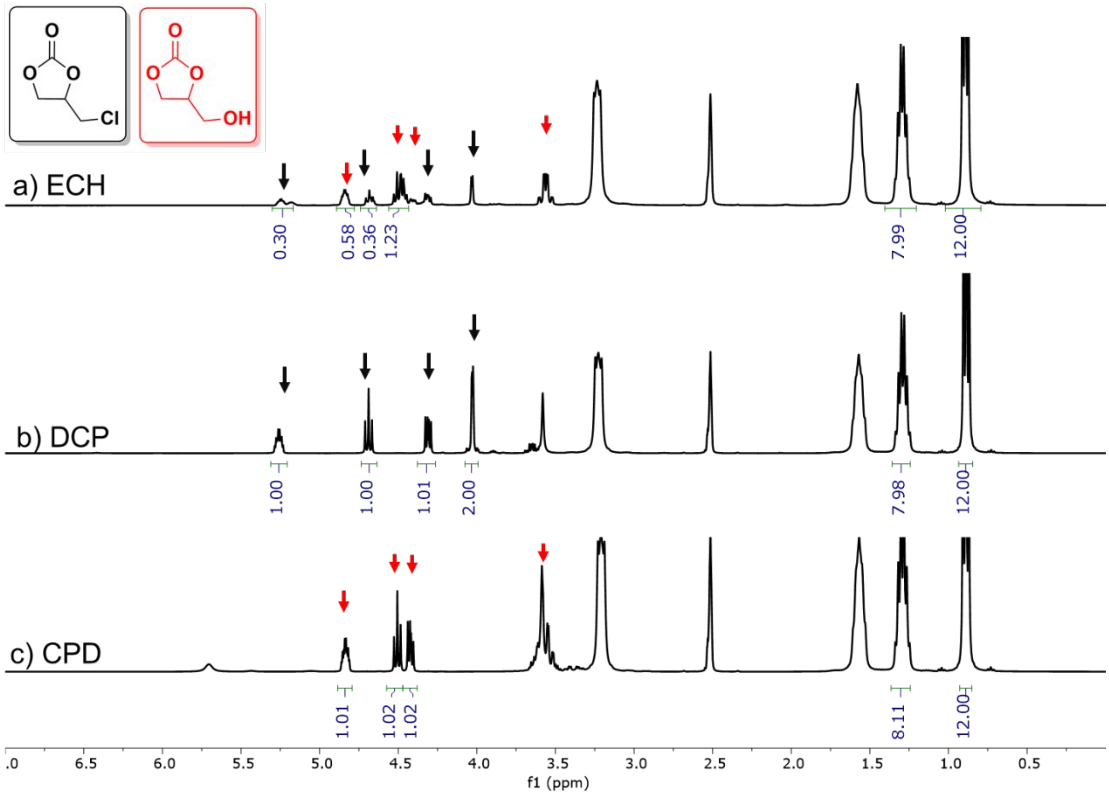


Figure S 23. $^{1}$H NMR spectra (400 MHz, 298 K) of cycloaddition reaction, substrate, TBA.OH (100 mol%), 16 h, $CO_2$ previously bubbled, 0.5 mL DMSO. (a) ECH; (b) DCP; (c) CPD.

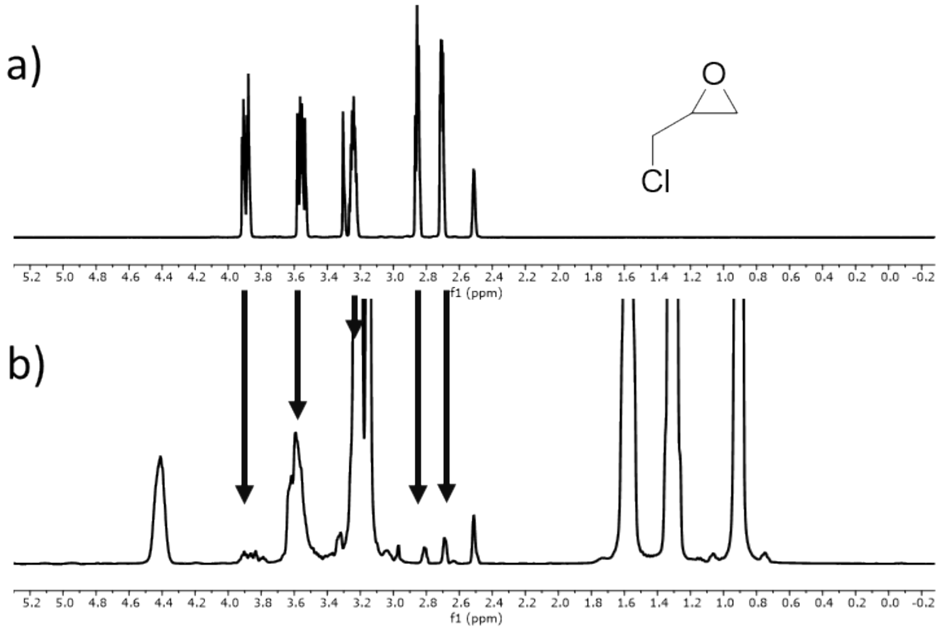


Figure S 24. $^{1}$H NMR spectra (400 MHz, 298 K) test of reaction without $CO_2$ to evaluate the mechanism: DCP: TBA.OH (1:1 Eq) in DMSO 40 $^oC$, 16h. (a) ECH neat; (b) reaction.

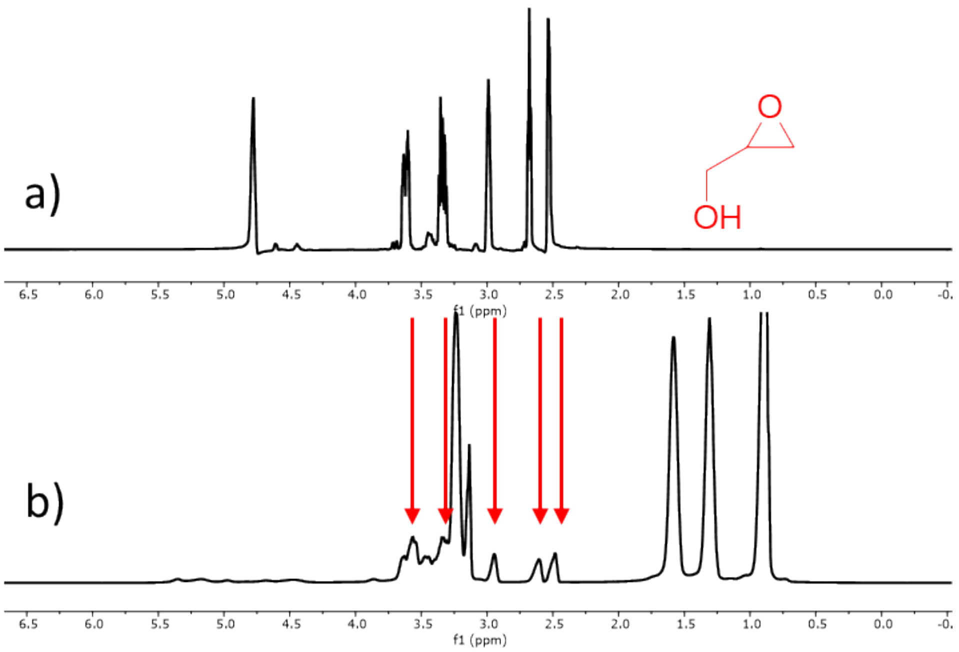


Figure S 25. $^{1}$H NMR spectra (400 MHz, 298 K) test of reaction without $CO_2$ to evaluate the mechanism: CPD: TBA.OH (1:1 Eq) in DMSO 40 °C, 16h. (a) glycidol neat; (b) reaction.

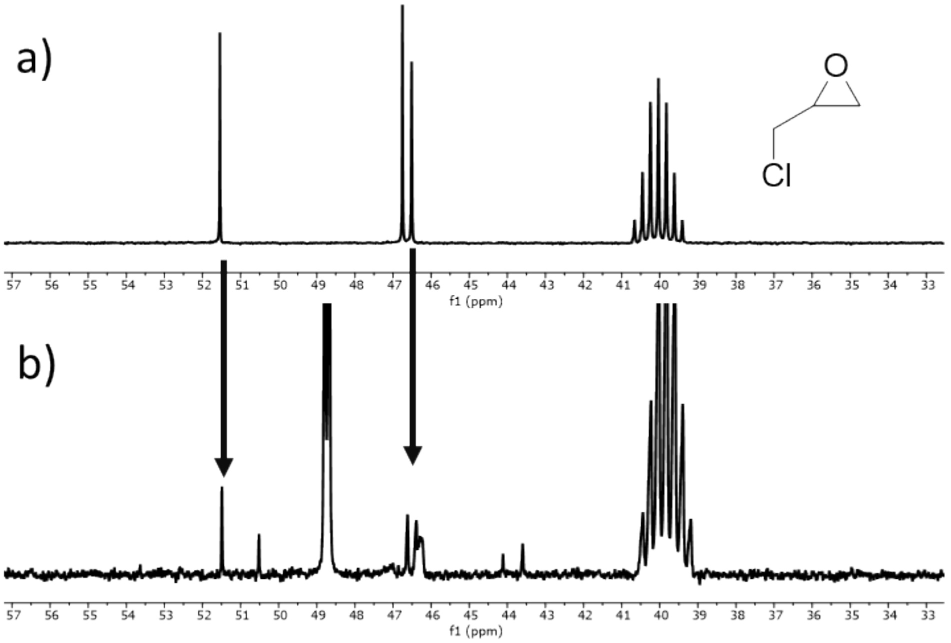


Figure S 26. $^{13}$C NMR spectra (100 MHz, 298 K) test of reaction without CO2 to evaluate the mechanism: DCP: TBA.OH (1:1 Eq) in DMSO 40 °C, 16h. (a) ECH neat; (b) reaction.

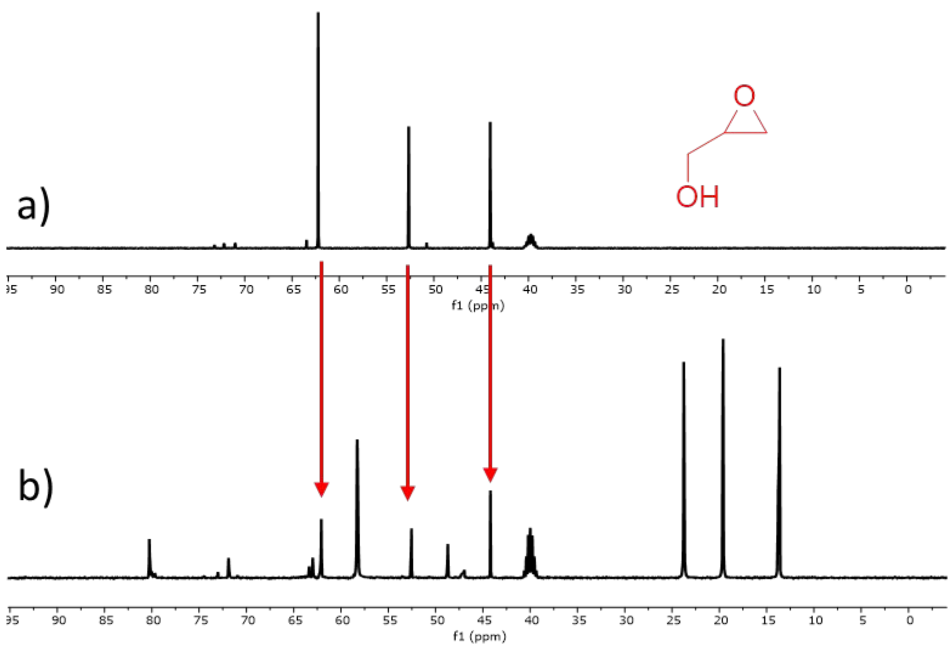


Figure S 27. $^{13}C$ NMR spectra (100 MHz, 298 K) test of reaction without CO2 to evaluate the mechanism: CPD: TBA.OH (1:1 Eq) in DMSO 40 $^{o}C$, 16h. (a) glycidol neat; (b) reaction.

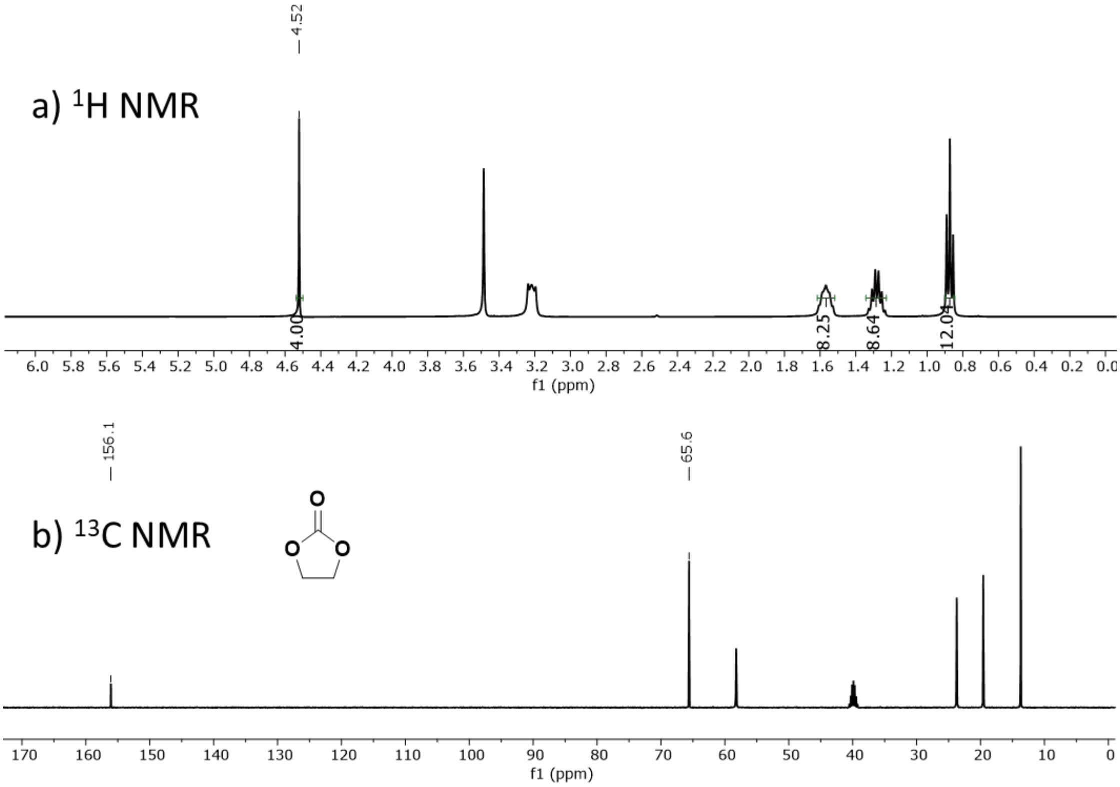


Figure S 28. NMR spectra (400 MHz, 298 K) of cycloaddition reaction to bromoethanol, TBA.OH (100 mol%), 16 h, $CO_2$ previously bubbled, 0.5 mL DMSO. (a) $^{1}H$ ; (b) $^{13}C$.

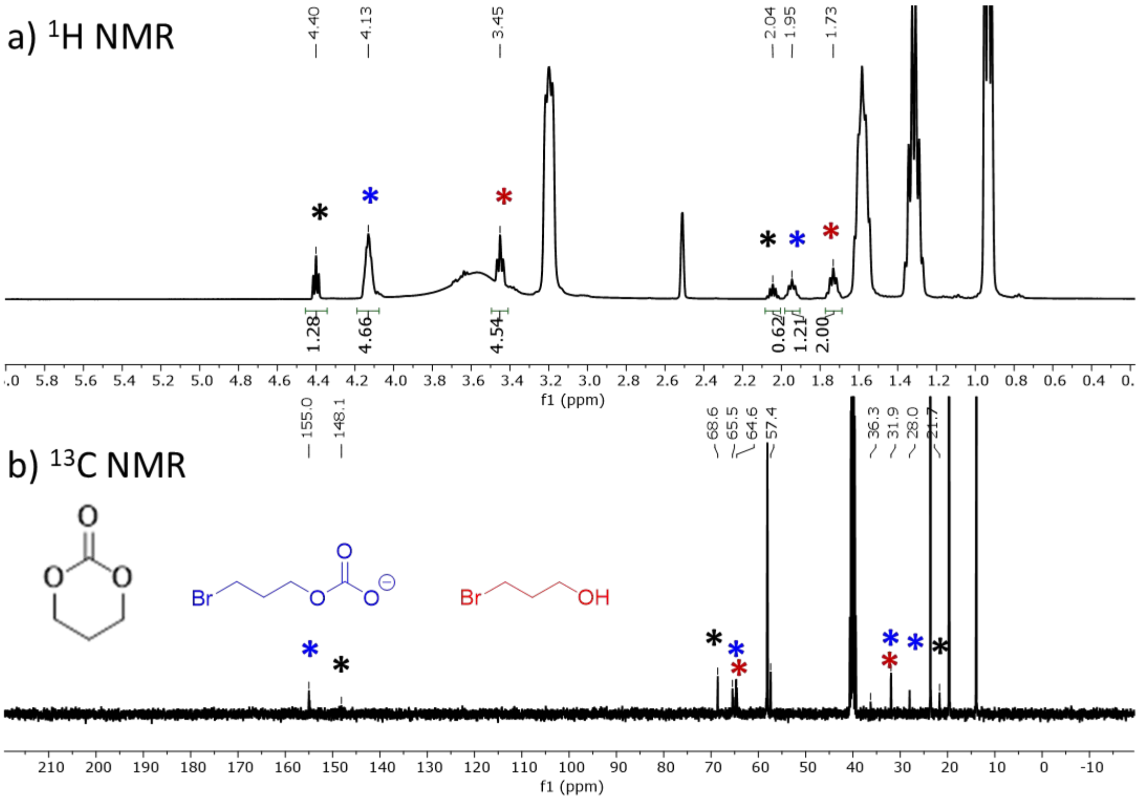


Figure S 29. NMR spectra (400 MHz, 298 K) of cycloaddition reaction to 1-bromopropan-3-ol, TBA.OH (100 mol%), 16 h, $CO_2$ previously bubbled, 0.5 mL DMSO. (a) $^{1}H$ ; (b) $^{13}C$.

## 4.2. Recycle experiments

A recharge experiment was performed bubbling more $CO_2$ until complete conversion of substrate, and a relation of 2 Eq of substrate to 1 Eq of IL were observed. The formed products present a relation of 1 Eq of GC and 1Eq of EC. With the increases of substrate amount the reaction do not occur anymore, and subproducts started to be formed (Figure S29)

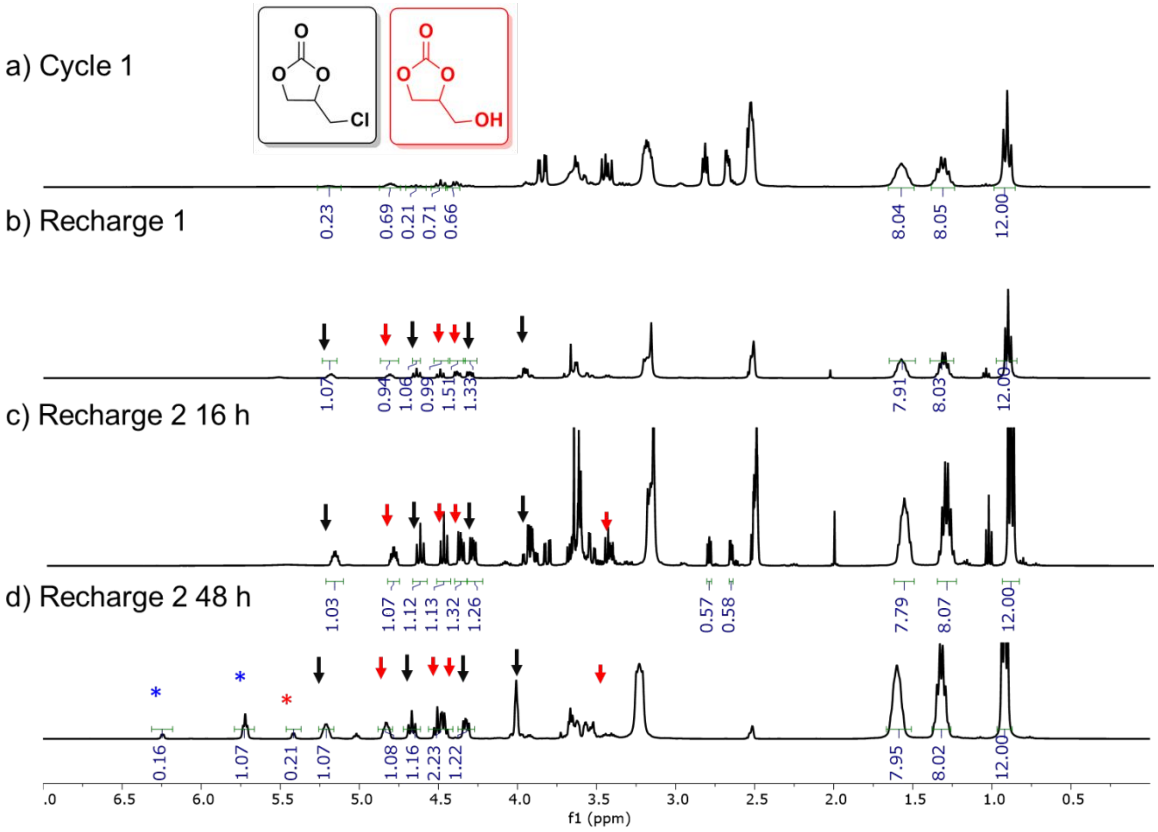


Figure S 30. {$^{1}$H} NMR spectra (400 MHz, 298 K) of cycloaddition reaction, substrate TBA.OH, 16 h, $CO_2$ previously bubbled, 0.5 mL DMSO. (a) Cycle 1 (100% conversion); (b) Recharge of ECH (16 h) – no increases in the conversion ; (c) Recharge of ECH (48 h) – degradation.

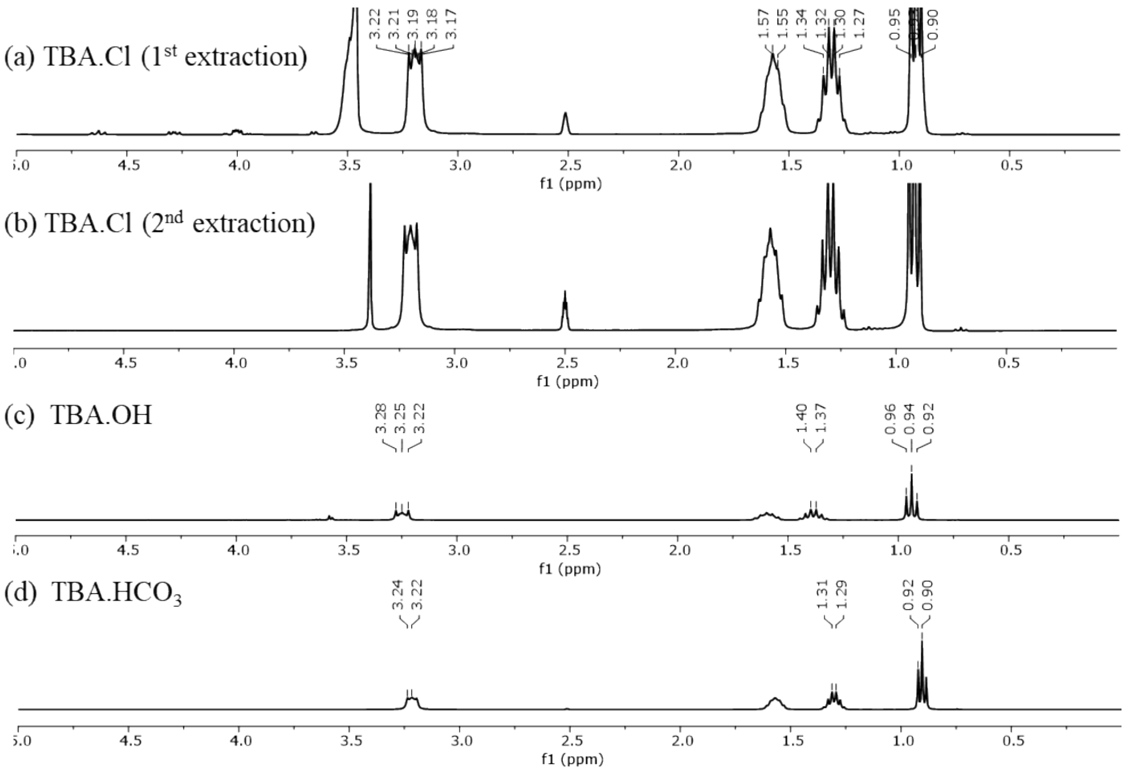

Figure S 31. $^1H$ NMR spectra (400 MHz, 298 K, DMSO) of TBA.X resultant of $CO_2$ cycloaddition reaction. (a) X= Cl, after diethyl acetate extraction. (b) X=Cl, after diethyl acetate and cold $CHCl_3$ extraction; (c) X= OH; after ion exchange column; (d) X=$HCO_3$, after atmospheric air bubbling for 16h.

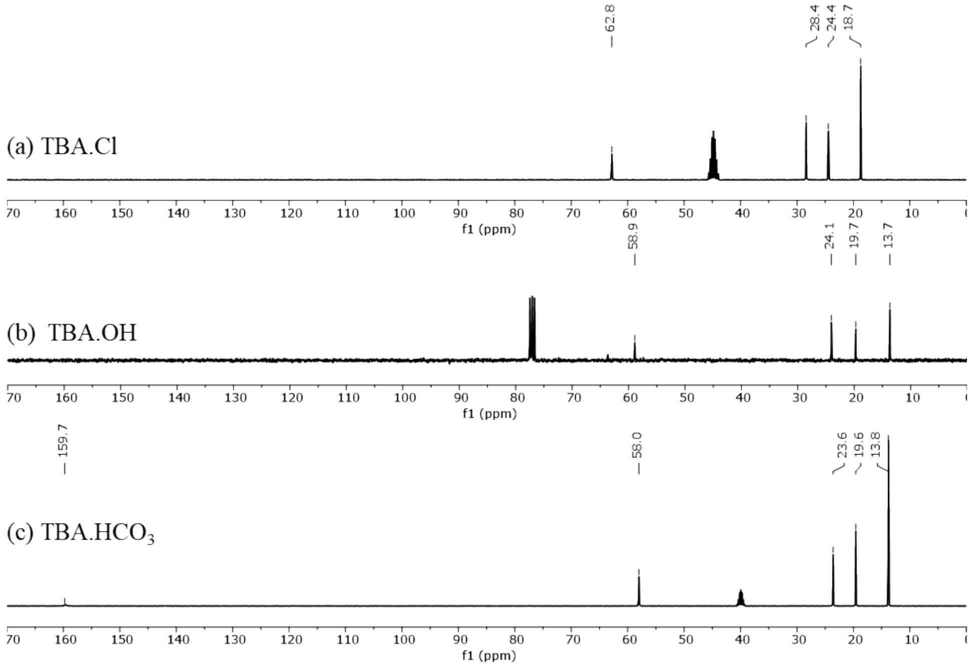


Figure S 32. $^{13}C$ NMR spectra (100 MHz, 298 K) of TBA.X resultant of $CO_2$ cycloaddition reaction. (a) X= Cl, after diethyl acetate and cold $CHCl_3$ extraction; (b) X= OH; after ion exchange column; (c) X=$HCO_3$, after atmospheric air bubbling for 16h.

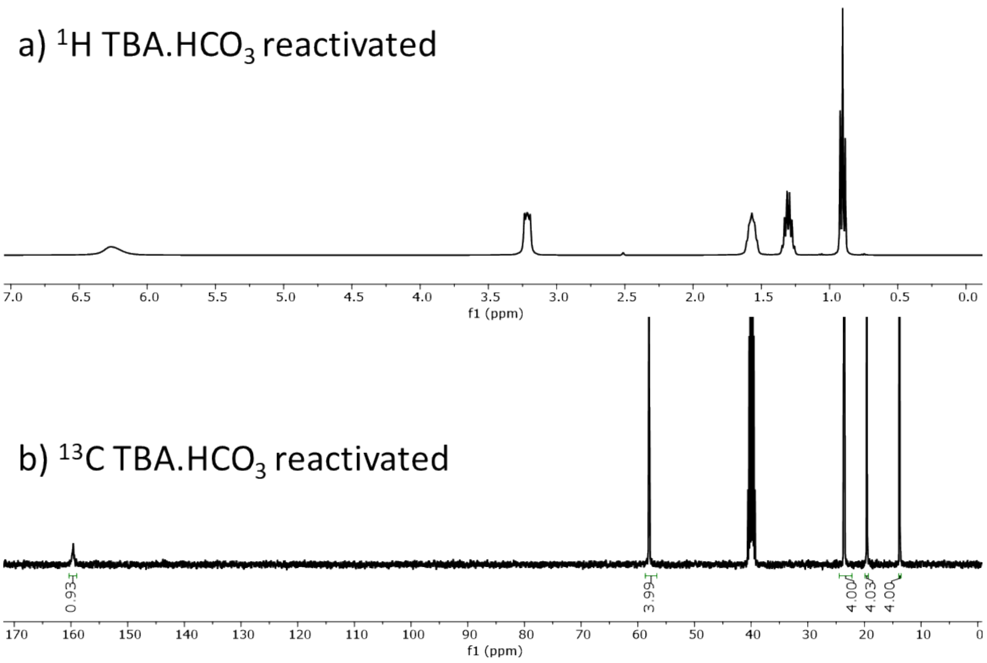


Figure S 33. NMR spectra (400 MHz, 298 K, DMSO) of TBA.$HCO_3$ after recycling (extraction; ion exchange column and bubbling atmospheric air in DMSO for 16h at room temperature). (a) $^1H$ NMR; (b) $^{13}C$ NMR.

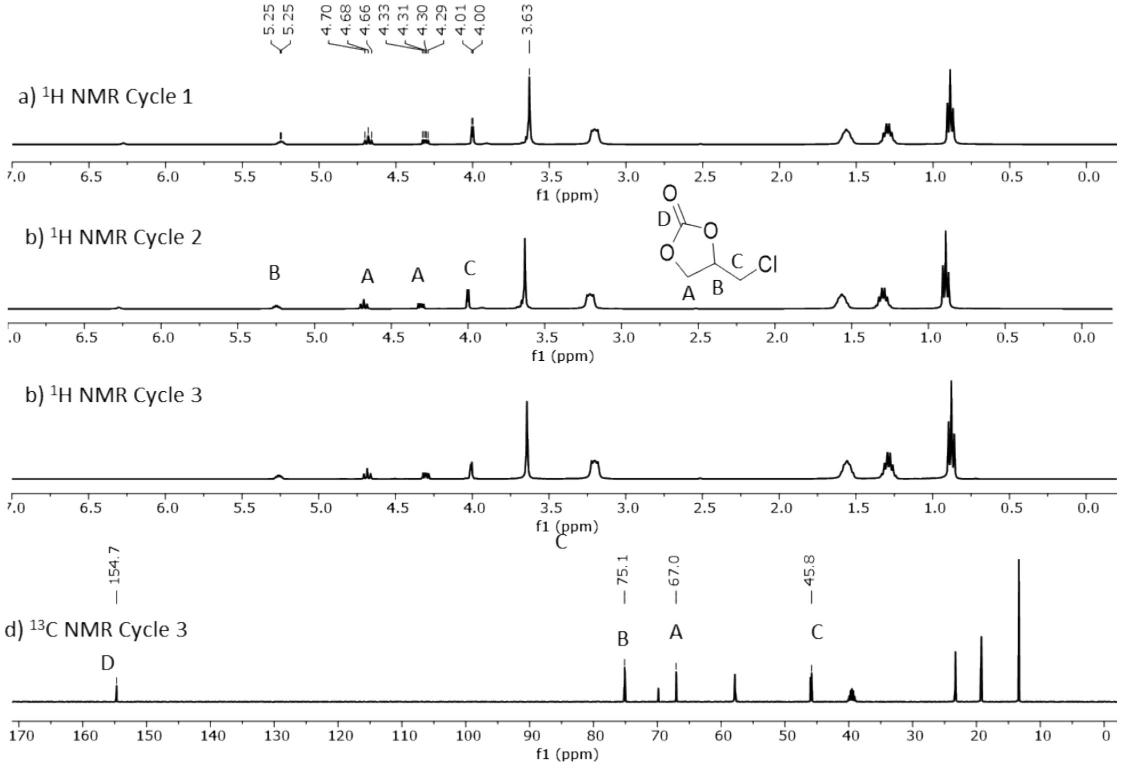


Figure S 34. NMR spectra (400 MHz, 298 K) of recycling reactions. (a) $^{1}$H NMR Cycle 1; (b) $^{1}$H NMR Cycle 2; (c) $^{1}$H NMR Cycle 3. (c) $^{13}$C NMR Cycle 3. *Reaction conditions*: DCP (1Eq), TBA.OH (1 eq) previously bubbled with atmospheric afir (16h rt), DMSO (0.5 mL) followed by addition of DCP (40$^{o}$C, 16 h, flow of atmospheric air).

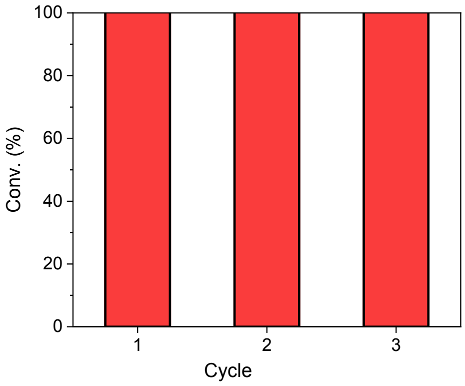


Figure S 35. Reuse of TBA.OH using DCP as substrate. *Reaction conditions: Step 1*: TBA.OH (0.5 mmol), DMSO or DMC (0.5 mL), previously bubbled compressed air (16 h, 25 $^{o}$C); Step 2: addition of substrate (0.5 mmol), 16 h, 40 ºC, atmospheric air flow.

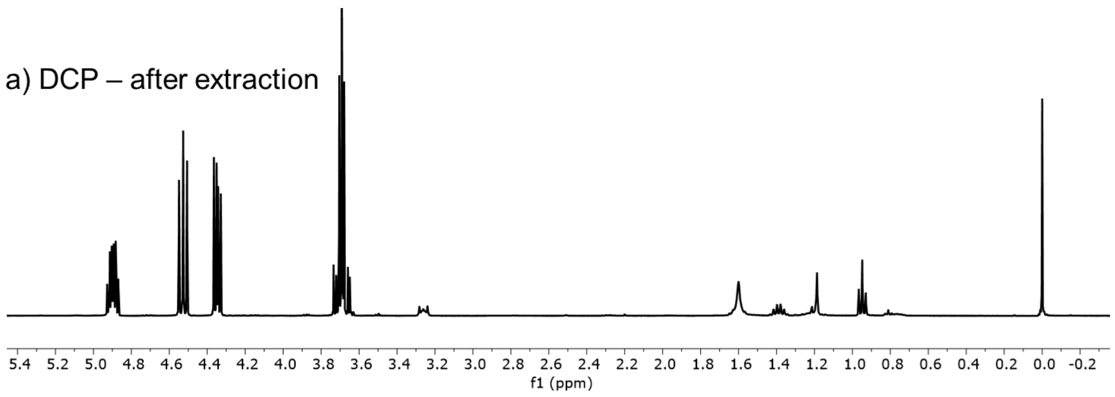


Figure S 36. $^{1}$H NMR spectra (400 MHz, 298 K) DCP extracted in the organic phase (see recycle methodology). Reaction cond.: TBA.OH, 16 h, $CO_2$ previously bubbled, 0.5 mL DMSO.

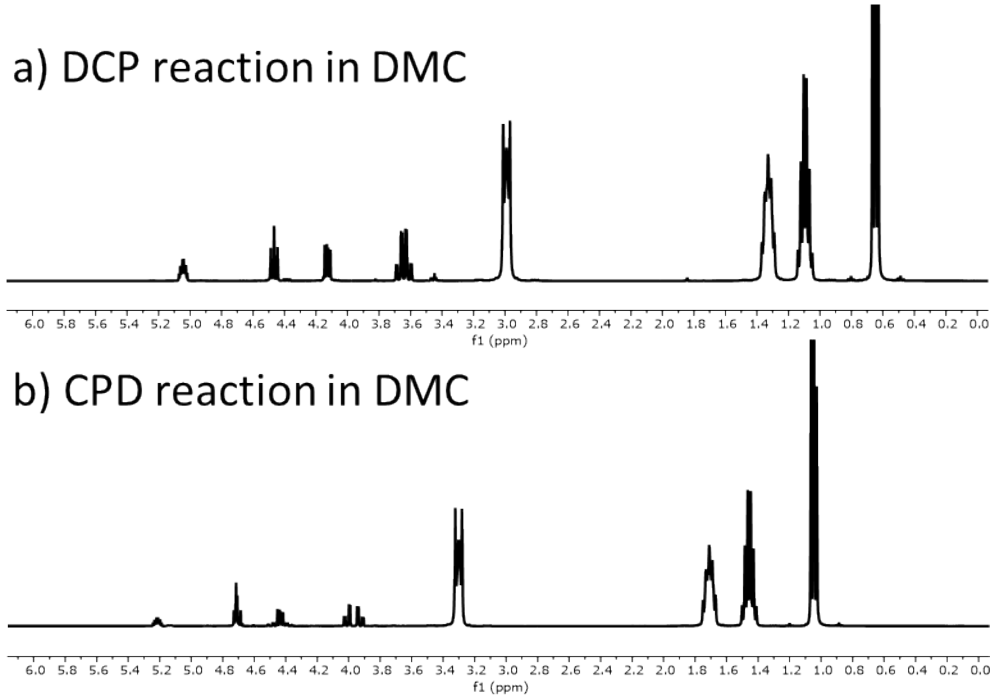


Figure S 37. $^{1}$H NMR spectra (400 MHz, 298 K) of cycloaddition reaction using halohydrin as substrate and DMC as solvent. *Reaction conditions: Step 1*: TBA.OH (0.5 mmol), DMC (0.5 mL), substrate. previously bubbled compressed air (16 h, 25 °C); Step 2: addition of substrate (0.5 mmol), 16 h, 40 °C, atmospheric air flow. A) DCP as substrate; B) CPD as substrate.

## 5. References


1 Corvo, M. C. *et al.* Solvation of Carbon Dioxide in [C4mim][BF4] and [C4mim][PF6] Ionic Liquids Revealed by High-Pressure NMR Spectroscopy. *Angewandte Chemie International Edition* **52**, 13024-13027, doi:https://doi.org/10.1002/anie.201305630 (2013).

2 Li, Z.-J., Sun, J.-F., Xu, Q.-Q. & Yin, J.-Z. Homogeneous and Heterogeneous Ionic Liquid System: Promising “Ideal Catalysts” for the Fixation of CO2 into Cyclic

Carbonates. *ChemCatChem* **13**, 1848-1866, doi:https://doi.org/10.1002/cctc.202001572 (2021).
3 Guo, L., Lamb, K. J. & North, M. Recent developments in organocatalysed transformations of epoxides and carbon dioxide into cyclic carbonates. *Green Chemistry* **23**, 77-118, doi:10.1039/D0GC03465G (2021).
4 Liu, L.-H., Liu, L., Chi, H.-R., Li, C.-N. & Han, Z.-B. A [(M2)6L8] metal–organic polyhedron with high CO2 uptake and efficient chemical conversion of CO2 under ambient conditions. *Chemical Communications*, doi:10.1039/D2CC01734B (2022).
5 Lv, H., Fan, L., Chen, H., Zhang, X. & Gao, Y. Nanochannel-based {BaZn}–organic framework for catalytic activity on the cycloaddition reaction of epoxides with CO2 and deacetalization-Knoevenagel condensation. *Dalton Transactions* **51**, 3546-3556, doi:10.1039/D1DT04231A (2022).
6 Pal, T. K., De, D. & Bharadwaj, P. K. Metal–organic frameworks for the chemical fixation of CO2 into cyclic carbonates. *Coordination Chemistry Reviews* **408**, 213173, doi:https://doi.org/10.1016/j.ccr.2019.213173 (2020).
7 Kumatabara, Y., Okada, M. & Shirakawa, S. Triethylamine Hydroiodide as a Simple Yet Effective Bifunctional Catalyst for CO2 Fixation Reactions with Epoxides under Mild Conditions. *ACS Sustainable Chemistry & Engineering* **5**, 7295-7301, doi:10.1021/acssuschemeng.7b01535 (2017).
8 Ge, Y., Cheng, G. & Ke, H. Triethanolamine borate as bifunctional Lewis pair catalyst for the cycloaddition of CO2 with epoxides. *Journal of CO2 Utilization* **57**, 101873, doi:https://doi.org/10.1016/j.jcou.2021.101873 (2022).
9 Castro-Osma, J. A., Lamb, K. J. & North, M. Cr(salophen) Complex Catalyzed Cyclic Carbonate Synthesis at Ambient Temperature And Pressure. *ACS Catalysis* **6**, 5012-5025, doi:10.1021/acscatal.6b01386 (2016).
10 Liu, K. *et al.* In situ synthesis of pyridinium-based ionic porous organic polymers with hydroxide anions and pyridinyl radicals for halogen-free catalytic fixation of atmospheric CO2. *Green Chemistry* **24**, 136-141, doi:10.1039/d1gc03465k (2022).
11 Xie, Y. *et al.* Hypercrosslinked mesoporous poly(ionic liquid)s with high ionic density for efficient CO2 capture and conversion into cyclic carbonates. *Journal of Materials Chemistry A* **6**, 6660-6666, doi:10.1039/C8TA01346B (2018).
12 Yang, X. *et al.* Deep Eutectic Solvents as Efficient Catalysts for Fixation of CO2 to Cyclic Carbonates at Ambient Temperature and Pressure through Synergetic Catalysis. *ACS Sustainable Chemistry & Engineering* **9**, 10437-10443, doi:10.1021/acssuschemeng.1c03187 (2021).
